# Weaving place‐based knowledge for culturally significant species in the age of genomics: Looking to the past to navigate the future

**DOI:** 10.1111/eva.13367

**Published:** 2022-04-08

**Authors:** Aisling Rayne, Stephanie Blair, Matthew Dale, Brendan Flack, John Hollows, Roger Moraga, Riki N. Parata, Makarini Rupene, Paulette Tamati‐Elliffe, Priscilla M. Wehi, Matthew J. Wylie, Tammy E. Steeves

**Affiliations:** ^1^ University of Canterbury School of Biological Sciences Christchurch New Zealand; ^2^ University of Otago Centre for Sustainability Dunedin New Zealand; ^3^ Te Rūnaka o Awarua Bluff New Zealand; ^4^ Waterscape Connections Ltd Dunedin New Zealand; ^5^ Te Rūnanga o Ngāi Tahu Dunedin New Zealand; ^6^ Kāti Huirapa Rūnaka ki Puketeraki Karitane New Zealand; ^7^ KEEWAI (Ernslaw One Ltd) Dunedin New Zealand; ^8^ Tea Break Bioinformatics Ltd Palmerston North New Zealand; ^9^ Hokonui Rūnanga Gore New Zealand; ^10^ 210125 University of Canterbury Ngāi Tahu Research Centre Christchurch New Zealand; ^11^ 210125 Environment Canterbury Christchurch New Zealand; ^12^ The New Zealand Institute for Plant and Food Research Limited Nelson New Zealand

**Keywords:** conservation, Indigenous knowledge, Indigenous Peoples and Local Communities, landscape genomics, local adaptation, local knowledge, mātauranga Māori, translocation

## Abstract

Relationships with place provide critical context for characterizing biocultural diversity. Yet, genetic and genomic studies are rarely informed by Indigenous or local knowledge, processes, and practices, including the movement of culturally significant species. Here, we show how place‐based knowledge can better reveal the biocultural complexities of genetic or genomic data derived from culturally significant species. As a case study, we focus on culturally significant southern freshwater kōura (crayfish) in Aotearoa me Te Waipounamu (New Zealand, herein Aotearoa NZ). Our results, based on genotyping‐by‐sequencing markers, reveal strong population genetic structure along with signatures of population admixture in 19 genetically depauperate populations across the east coast of Te Waipounamu. Environment association and differentiation analyses for local adaptation also indicate a role for hydroclimatic variables—including temperature, precipitation, and water flow regimes—in shaping local adaptation in kōura. Through trusted partnerships between community and researchers, weaving genomic markers with place‐based knowledge has both provided invaluable context for the interpretation of data and created opportunities to reconnect people and place. We envisage such trusted partnerships guiding future genomic research for culturally significant species in Aotearoa NZ and beyond.

**Positionality statement and terminology**

We, the authorship team, give thanks to the past, present, and future generations of Kāi Tahu, Kāti Mamoe, and Waitaha whānui (a collective of tribal groups in Te Waipounamu) and local communities that have guided the conception and writing of this manuscript. Our authorship includes Kāi Tahu researchers and tākata tiaki (guardians) Steph Blair (Kāi Tahu, Kāti Māmoe, Waitaha), Brendan Flack (Kāi Te Ruahikihiki, Kāi Tahu), Riki Parata (Ngāi Tahu, Te Atiawa, Ngāti Toarangatira, Ngāti Ruanui), Makarini Rupene (Ngāi Tūāhuriri, Ngāi Tahu), Paulette Tamati‐Elliffe (Kāi Te Pahi, Kāi Te Ruahikihiki (Ōtākou), Te Atiawa, Ngāti Mutunga), and Dr Matthew Wylie (Kāti Huirapa ki Puketeraki, Kāi Tahu). Roger Moraga, of Spain, is a research bioinformatician. The remaining authors are Pākehā (New Zealander of European descent) researchers and practitioners: Dr Aisling Rayne and John Hollows are New Zealand Pākehā; Matthew Dale is Australian Pākehā from Gunaikurnai Country; Dr Priscilla Wehi is New Zealand Pākehā with familial affiliations to Waikato‐Tainui and Ngāpuhi; and Dr Tammy Steeves is Canadian Pākehā. Our collective expertise includes aquaculture, bioinformatics, customary and contemporary mahika kai (food gathering including processes, practices, and places), conservation genomics, fish biology, ecology, and te reo Māori (Māori language).

Herein, we use the Kāi Tahu dialect *k* in place of the northern *ng* (underlined in text) unless deemed inappropriate (e.g., quoted text, proper nouns, and particular papatipu rūnaka dialect). This reflects local pronunciation and does not necessarily change the meaning of the word (i.e., where underlined, *ng* and *k* are interchangeable). For non‐English words or phrases, definitions are provided in brackets at their first mention. A brief glossary for frequently used Māori terms is also provided in Appendix 1, although it does not capture their full or exhaustive meanings. We use worldview to refer to people's understanding of their relationship with the world, and we use knowledge to refer to knowledge systems and epistemologies, while acknowledging the limitations of this terminology (Berkes, 2009; Kimmerer, 2013). We refer to knowledge and methodologies embedded in neoclassical traditions as Western science.

## 1 INTRODUCTION

From the deep past to the present, Indigenous and local relationships with place have long been intertwined with those of non‐human biodiversity (Watts, 2013), including through movement (*translocation*) of plants and animals (Hamley et al., 2021; Turner et al., 2000). Such place‐based relationships shape language, practices, and processes passed down generations (Black, 2014; Nazarea, 2006; Wehi et al., 2020). In turn, those relationships are encoded into the DNA of plants and animals (Matisoo‐Smith, 2007; Silcock, 2018). Western‐trained scientists increasingly recognize that genetic and genomic research involving species treasured by Indigenous Peoples and Local Communities (IPLC; Reyes‐García et al., 2019) sits at the interface of biological, cultural, and linguistic (i.e., *biocultural)* systems (Bridgewater & Rotherham, 2019). Yet, in practice, few studies incorporate the contextual fabric of Indigenous and local worldviews that give genomic or ecological data meaning. Similarly, few consider how the processes of gathering, interpreting, and sharing those data can reconnect people and places.

Genetic and genomic data map connections between individuals, populations, and/or the environment (Manel et al., 2003). These connections can be used to examine past and present patterns, and to co‐develop strategies that increase species’ resilience to future challenges (Frankham et al., 2019; Hohenlohe et al., 2021). For example, increased research capacity and capability to characterize genomic markers (e.g., single‐nucleotide polymorphisms, SNPs) under selection (*adaptive variation*) is informing how populations are prioritized for conservation (e.g., Barbosa et al., 2018; Funk et al., 2012; Harrisson et al., 2017), including decisions around whether or how to translocate (e.g., Capel et al., 2021; Furlan et al., 2020; MacLachlan et al., 2021; Robinson et al., 2021). However, testing relationships between genetic variation and fitness across time and complex spatial landscapes is challenging, especially for widely distributed, non‐model animal species (e.g., Liddell et al., 2020; Seaborn et al., 2021). These relationships are further complicated by diversity in genomic architecture (e.g., in copy number variation, chromosome inversions, and transposable elements; Dorant et al., 2020; Wellenreuther et al., 2019; Wold et al., 2021) and by sources of adaptive potential that extend into the realm of transcriptomics (e.g., Oostra et al., 2018). Although researchers should avoid overpromising in the face of these complexities (Kardos et al., 2021; Parker et al., 2020 *preprint*), we still consider it useful to explore how characterizing adaptive variation could support conservation decisions, especially for fragmented species facing rapid environmental change (e.g., Brauer & Beheregaray, 2020; Eikaas & McIntosh, 2006). In the next section, we consider how trusted relationships between researchers and IPLC could guide the co‐creation of genomic research situated in biocultural context.

### 1.1 Place‐based knowledge provide biocultural context for genetic and genomic data

Genetic and genomic studies have long recognized how complex evolutionary dynamics, such as gene flow, shape genetic diversity, including local adaptation (e.g., Attard et al., 2021; Beheregaray & Caccone, 2007; Ralls et al., 2018; Tigano & Friesen, 2016). For culturally significant species, these dynamics are often linked to IPLC knowledge and practices. For example, in Te Waipounamu, the South Island of Aotearoa NZ, Kāi Tahu ancestors carefully managed many species and landscapes. Tau et al. (1990) reflect that,

Shellfish beds were seeded with superior strains taken from other areas, and established beds were both enhanced and depleted by biological methods. Stands of trees such as karaka (*Corynocarpus laevigatus*) and tī kōuka (*Cordyline australis*, cabbage tree) were planted from selected stock and were actively managed to optimize their production. Examples of these managed plantings can still be seen in the contemporary landscape.

Yet, place‐based knowledge, such as human‐mediated translocations, remains strikingly absent from many efforts to characterize and interpret genetic variation (although see Einfeldt et al., 2020; Sutherland et al., 2020). In contrast, recent genetic or genomic studies led by, co‐led by, or involving IPLC (for example, see Bowles et al., 2020; Garner et al., 2016; Gros‐Balthazard et al., 2020; Henson et al., 2021; Polfus et al., 2016; Ross et al., 2018; Service et al., 2020) signal a shift toward co‐created biocultural and/or ethnobiological approaches. Such approaches may include exploration of oral traditions (e.g., Gros‐Balthazard et al., 2020; Wehi et al., 2009) and recognition of place‐based knowledge encoded in narrative, language, and through hunting, farming, or other practical experience (e.g., walking the land). For example, to ensure the sustainable supply of traditional weaving plants, Harris et al. (2005) assessed plant growth and susceptibility to cold damage in harakeke and wharariki (*Phormium tenax*, and *P*. *cookianum*, respectively) through common garden experiments involving local weavers, teachers, and students. Beyond their extensive ecological and cultural knowledge of these species, participants “*provided land for the plantings*, *assisted in maintenance and measurement*, *and fostered extension and educational use of the plantings*” (Harris et al., 2005).

There is a breadth of benefits that accompany co‐created approaches to genomic research. These benefits may include but are not limited to: (1) Reciprocally enhanced understanding of complex biocultural histories through genomics and/or place‐based knowledge, including the co‐production of *a priori* hypotheses. For instance, a recent collaboration between Nuxalk, Haíɫzaqv, Kitasoo/Xai'xais, Gitga'at, and Wuikinuxv First Nations and conservation scientists found convergence between grizzly bear *Ursus arcto*s genetic and human linguistic diversity, suggesting that human groups and grizzly bears have been shaped by the landscape in similar ways (Henson et al., 2021). In another recent study, integrating ethnographic survey (i.e., interviews and observation of farming practices) and population genetic analysis promoted a richer understanding of date palm *Phoenix dactylifera L*. diversity in Siwa Oasis, Egypt (Gros‐Balthazard et al., 2020). For example, Isiwan farming practices and local ways of classifying biodiversity enabled the formation of hypotheses and sampling strategies to assess crop evolution and diversity and verified molecular genetic analyses, which in turn, resolved gaps or uncertainties in local knowledge; (2) Improved quality and breadth of genomic data through opportunities to collect fresh tissue or blood, to access restricted or private sites, or to include treasured samples or populations with unique histories (e.g., Collier‐Robinson et al., 2019); (3) Improved selection and quality of environmental predictors and fitness parameters in efforts to characterize local adaptation (e.g., for environment‐association analyses, EAAs, or genome‐wide association studies, GWAS). Namely, IPLC have identified measures of fitness poorly defined in Western scientific literature (e.g., sexual dimorphism in kōkō or tūī *Prosthemadera novaeseelandiae*; Wehi et al., 2019), including culturally desirable characteristics (e.g., superior fruit load, size, and sweetness in wild loquat *Uapaca kirkiana* in Malawi and Zambia; Mng’omba et al., 2015). Place‐based knowledge can also reveal fine‐scale, stochastic, or changing selection pressures not readily captured by climatic (e.g., temperature and precipitation) or macro‐ecological predictors such as elevation (Herse et al., 2020; Hoban et al., 2016). For example, the contemporary distribution of karaka translocated and cultivated by Māori (Atherton et al., 2015) and culturally significant medicinal and weaving plants experiencing rapid climate‐driven shifts (Bond et al., 2019) may poorly reflect past selection pressures; and (4) Enhanced understanding of how selection pressures interact with traits of interest. For instance, in Lyver et al. (2009), elders of Tūhoe (a Māori tribe in Te Urewera region of Aotearoa NZ) describe how climatic warming has impacted the nutrition and body condition of kererū *Hemiphaga novaeseelandiae* through increasingly delayed fruiting of trees such as toromiro *Podocarpus ferrugineus*.

**FIGURE 1:**
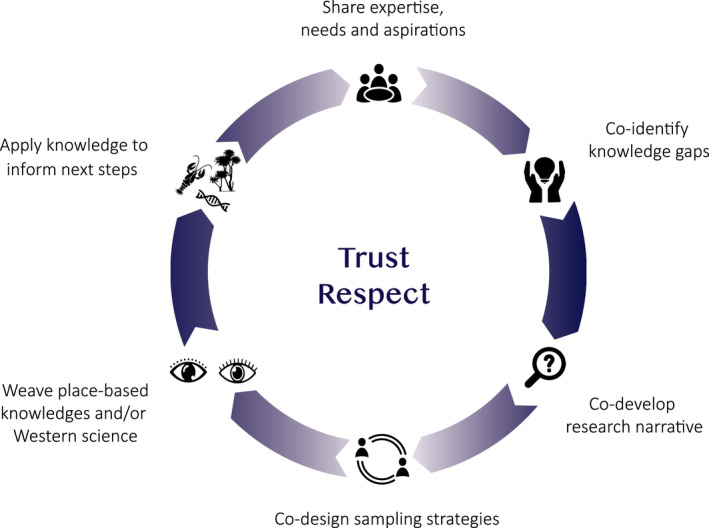
An illustration of our approach to building research partnerships that weave place‐based knowledge and genomic data to enhance the characterization of biocultural diversity in freshwater kōura *(Paranephrops zealandicus)* in Te Waipounamu, Aotearoa NZ. Research partnerships are built on mutual trust and respect with opportunities to grow capability and capacity among all partners. This framework is adapted from the graphical representation of the multiple evidence‐based approach (Tengö et al., 2014) presented in Tengö et al. (2017) and the knowledge co‐evolution framework presented in Chapman and Schott (2020)

Amid growing initiatives to realize Indigenous rights and interests in genomic resources including data derived from culturally significant species (Handsley‐Davis et al., 2020; Hudson et al., 2020), there is every reason to co‐create genomic research that reflects place‐based relationships and diverse aspirations. Below, we present a framework adapted from extensive scholarship (e.g., Chambers et al., 2021; Chapman & Schott, 2020; Claw et al., 2018; Hudson et al., 2020; Tengö et al., 2014, 2017) to illustrate our approach to building research partnerships for a culturally significant species in Te Waipounamu, Aotearoa NZ (Figure 1). In the next section, we walk through this framework step‐by‐step to show how we have woven place‐based knowledge with genomic markers in ways responsive to IPLC aspirations.

### 1.2 A case study: first steps to weaving place‐based knowledge with a genomic study of kōura in Te Waipounamu, Aotearoa NZ

After several thousand years’ navigating the Pacific Ocean, the Polynesian ancestors of Māori first settled in Aotearoa NZ around 800 years ago. Māori were followed by European colonizers in the late 18^th^ century and people from throughout the world since then (O’Malley, 2013; Wilmshurst et al., 2011). The long affiliations between Māori and the land and waterscapes of the Pacific are mapped and layered through *whakapapa*, here referring to the physical and metaphysical connections, lineages, and genealogical systems that connect all things across time and place (Tau, 2001; Te Rito, 2007). As Roberts (2013) describes, “*whakapapa as a philosophical construct implies that all things have an origin … and that ontologically things come into being through the process of descent from an ancestor or ancestors*.*”* Thus, it is whakapapa that gives context and relevance to Māori socio‐environmental ethics (Harmsworth & Awatere, 2013; Kawharu, 2000), including through empirically derived biological knowledge and accompanying narratives (Roberts, 2013; Roberts et al., 2004). In the context of genomic research, any DNA—and by extension, genomic data—can be considered a physical form of whakapapa (Collier‐Robinson et al., 2019). As such, the principles, rights, and responsibilities associated with whakapapa should guide whether and how genomic research involving species treasured by Māori is conducted (Collier‐Robinson et al., 2019; Hudson et al., 2021; Roberts et al., 2004).

In Te Waipounamu, the South Island of Aotearoa NZ, the whakapapa of many treasured species and places is interwoven with the processes and practices of mahika kai, described as “*the customary gathering of food and natural materials and the places where those resources are gathered*” (Ngāi Tahu Settlement Claims Act 1998; also see Phillips et al., 2016). Literally translated in English as “working the food”, mahika kai links people to their environment through language, practices, and processes and embodies reciprocity and responsibility toward others, including the natural environment. For Kāi Tahu, a tribal group in Te Waipounamu, mahika kai was—and continues to be—integral to the tribe's way of life. Rights and access to mahika kai were severely disrupted from the early 19^th^ century as European colonists (Pākehā) sought to dispossess Kāi Tahu and other tribal groups of their land, language, and practices. In turn, Pākehā and other non‐Māori brought their own aspirations and ways of managing the environment that continue to shape Aotearoa NZ’s land and waterscapes today.

Southern kōura *Paranephrops zealandicus* (White, 1847), found along the eastern and southern parts of Te Waipounamu, are one of two species of freshwater crayfish endemic to Aotearoa NZ (Hopkins, 1970) that play an important role in customary and contemporary mahika kai (Hiroa, 1921; Parata, 2019; Williams et al., 2017). Culturally and economically important to Māori and non‐Māori, they are known by various names including kēkēwai, wai kōura, kēwai, and crawlies. Here, we refer to kōura to reflect the paper's wider authorship but acknowledge kēkēwai as the name preferentially used by our Ngāi Tūāhuriri research partners. Environmental stressors associated with land‐use change are driving many kōura populations to extirpation (listed by the Department of Conservation as *At Risk*: *Declining*; Grainger et al., 2018; Kelly, 2019). Although excellent generalists (Whitmore et al., 2000) and ecosystem bioengineers (Parkyn et al., 1997), they are vulnerable to declining water quality, habitat loss, and introduced predators (Grainger et al., 2018; Parata, 2019; Thoms, 2016). Like many native freshwater crayfish elsewhere (e.g., Hossain et al., 2018; Whiterod et al., 2017), low dispersal capacity and slow reproductive rates have restricted their capacity to recolonize waterways following local extirpation (Fordham et al., 1979). Today, their distribution is increasingly fragmented, especially in heavily modified landscapes such as the Waitaha plains (Thoms, 2016).

### 1.3 Sharing expertise, needs, and aspirations

There are presently few published resources for kōura beyond the KEEWAI farming manual (Hollows, 2016), a handful of ecological studies (e.g., Kusabs et al., 2018; Whitmore et al., 2000), a recent transcriptome assembly (Oliphant et al., 2020), and a phylogeographic study based on a single mitochondrial marker (Apte et al., 2007). However, place‐based knowledge of kōura and other mahika kai species is maintained through narratives, practical experience, tribal archives, and other knowledge passed down generations. Recent interest in kōura has presented opportunities to build or strengthen relationships between whānau (family groups), papatipu rūnaka (local tribal groups with guardianship over land and water within their territory), practitioners, and researchers through place‐based approaches. Namely, there is growing interest in better understanding the whakapapa of populations—especially in relation to ancestral pathways—to enhance environmental well‐being and to reconnect people and place, including through the revitalization of sustainable harvest.

Below, we present genotyping‐by‐sequencing data for 19 kōura populations across Te Waipounamu and the ways in which we are weaving these data with place‐based knowledge. In this study, we focus on contemporary translocations and population genetic structure to provide a foundation for future research that will incorporate more comprehensive genomic sampling and place‐based knowledge. We also use redundancy analysis (RDA) to assess whether reduced representation genomic markers are useful for characterizing local adaptation in kōura. Specifically, we aimed to:

1. Examine genome‐wide diversity in kōura informed by place‐based knowledge;
2. Assess the benefits and challenges of characterizing local adaptation in kōura; and
3. Consider how these data can inform the management of kōura in the Kāi Tahu region to meet mahika kai aspirations, including enhanced biocultural resilience.

## 2 MATERIALS AND METHODS

We preface this study by acknowledging that requirements and processes for ethical research engagement vary across communities, institutions, and contexts. At the time of this study, there was no formal requirement nor procedure for researchers working with culturally significant species to engage with Māori beyond the responsibilities articulated in Te Tiriti o Waitangi (1840)—which guarantees Māori self‐determination, including sovereignty over their treasured possessions, culture, and species—and the WAI 262 Waitangi Tribunal claim (1991) and subsequent Ko Aotearoa Tēnei report (Waitangi Tribunal, 2011).

### 2.1 Framing the research narrative

**FIGURE 2:**
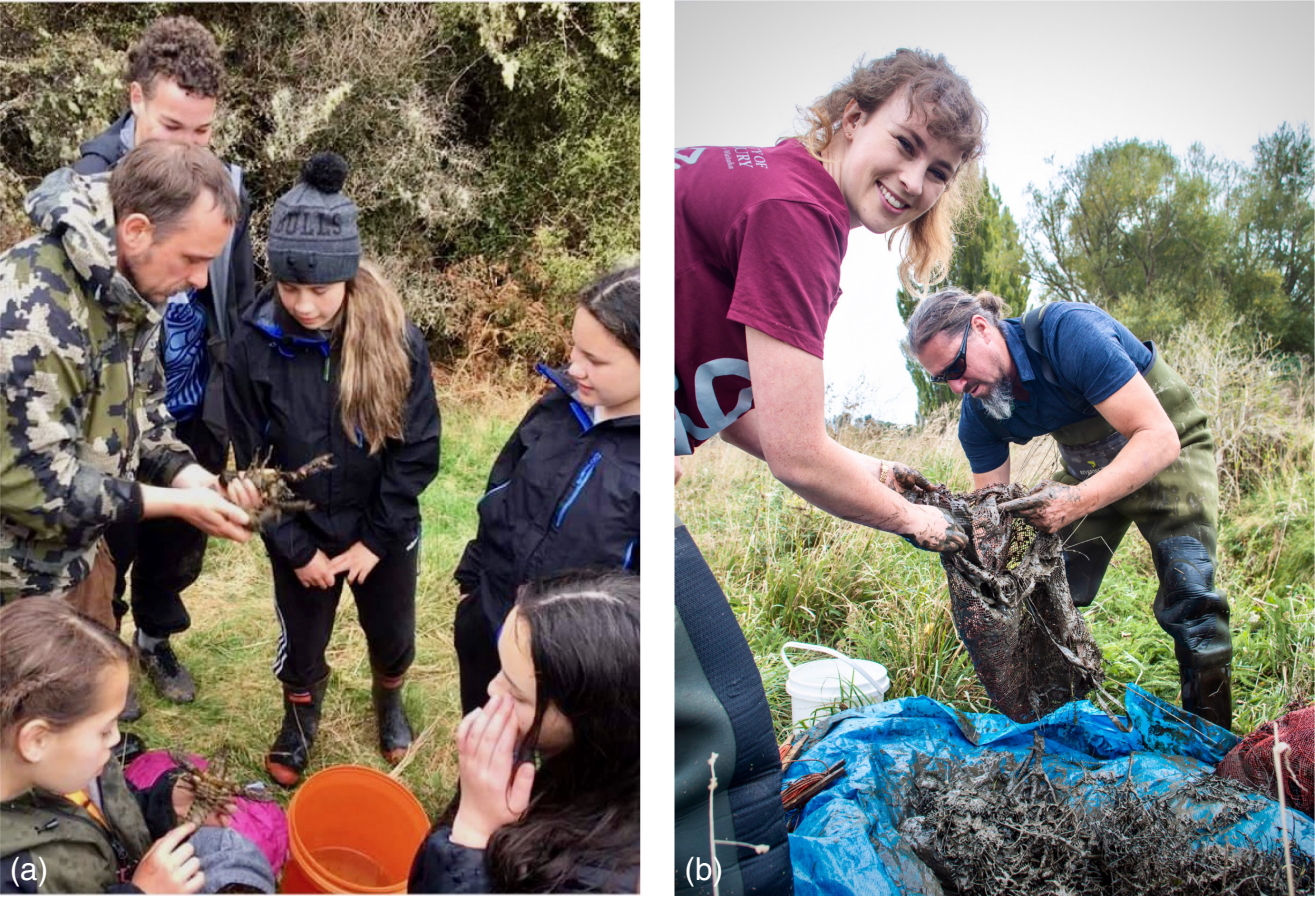
Sampling kōura for DNA sequencing. (a) Co‐author Matt Dale with rakatahi (youth) sampling kōura on a Rangatahi Tumeke camp in Murihiku. (b) Co‐authors Aisling Rayne (left) and Makarini Rupene (right) finding an individual for the reference genome, using modified whakaweku (bracken fern bundles) at a site significant to Ngāi Tūāhuriri

Our research narrative begins with a dialogue between researchers at the University of Canterbury and Ngāi Tūāhuriri, a Kāi Tahu subtribe with local authority. From those early conversations, Ngāi Tūāhuriri determined that conservation genomics research could be of benefit and identified kōura as a culturally significant species in need of further research. Together, we co‐developed an iterative timeline that upholds the decision‐making authority of Ngāi Tūāhuriri at each research step, including data generation, storage, and access (see Collier‐Robinson et al., 2019). During these years, other partnerships were developed beyond the territory of Ngāi Tūāhuriri, including with whānau from Wairewa, Te Taumutu Rūnanga, Kāti Huirapa Rūnaka ki Puketeraki, Te Rūnaka o Ōtākou, Kāi Tahu ki Murihiku (Waihōpai Rūnaka, Awarua Rūnanga, Ōraka‐Aparima Rūnaka, and Hokonui Rūnanga), and KEEWAI. In general, these partnerships began with a kanohi‐ki‐te‐kanohi (‘face‐to‐face”) conversation. For example, the inclusion of a Murihiku site originated through a serendipitous meeting between co‐authors Aisling Rayne and Matthew Dale at a Wairewa cultural monitoring wānaka (learning forum) for tuna (eel *Anguilla spp*). This meeting led to subsequent conversations, including eventual introductions between Rayne and Murihiku whānau. A year later, co‐authors Dale and Steph Blair identified an opportunity for Rayne to attend a Rangatahi Tumeke camp (Figure 2a), where cultural experts and rakatahi (Māori youth) come together to reconnect with mahika kai.

Before, during, and after sampling, the benefits and risks of examining genetic diversity in kōura were discussed among researchers, practitioners, and tribal representatives. We discussed how this work, together with oral histories of translocation, could help whānau and local communities navigate conservation policy and regulations (for example, to revitalize movement between catchments or re‐establish locally extirpated populations; Dale, Hollows, pers. obs.). It was also critical to ensure that opportunities and risks related to generating, storing, and accessing culturally significant genomic data were understood by all partners, including options for data storage that extend beyond open access.

### 2.2 Co‐design of sampling strategies

Through a combination of place‐based knowledge, grey literature, and publicly available distribution records, we identified and sampled 19 sites in the territory of Kāi Tahu across three main regions: Waitaha, Ōtākou, and Murihiku. GPS coordinates are not presented here given the cultural significance of sampling sites. Most sites were identified by tākata tiaki (guardians) or local whānau and, where possible, we involved rakatahi (Māori youth) in sampling. Kōura were only retrieved from four sites in Waitaha due to their increasingly sparse distribution and from six sites in Murihiku where relationships were developed relatively late in the study.

During sampling, our partnerships ensured that relevant local or cultural protocol was followed. Practitioners and whānau identified sites unrecorded in public databases and selected appropriate sampling strategies for the habitat and people involved. These methods ranged from collection by hand, night spotlighting, gee‐minnow traps, and electric fishing to traditional or modified whakaweku (bracken fern bundles; Kusabs & Quinn, 2009). For example, to collect kōura for the short‐read reference genome (Section 2.3, below), a culturally significant site (W4) was identified by co‐author Makarini Rupene, kaitiaki mahika kai (customary food gathering expert) for Ngāi Tūāhuriri. Here, baited whakaweku were set and left for several weeks. When the maramataka (Māori lunar calendar; Roberts et al., 2006) indicated that kōura were active, we returned to retrieve the whakaweku (Figure 2b). To collect tissue samples, one pleopod (the third) was removed from each individual and stored in 100% ethanol under a University of Canterbury Animal Ethics Committee Permit (REF:2018/04R) before kōura were released back to the original sampling location.

### 2.3 Building a reference genome for kōura

To assemble a *de novo* short‐read reference genome, in April 2018, one male individual from site W4 (see Section 2.2) was immediately euthanized by placement in a −80°C bio‐freeze container (Bio‐Bottle™) as per Animal Ethics Committee approval (REF: 2018/04R). High‐molecular‐weight genomic DNA was extracted from tail muscle tissue at the University of Canterbury using the MagJET Magnetic Bead‐Based Nucleic Acid Purification kit (Thermo Fisher Scientific) as per protocol.

Three libraries of 2x 150 bp reads were prepared at the Institute of Clinical Molecular Biology (Kiel University) and sequenced with the Illumina HiSeq 4000. FastQC v. 0.11.8 (Andrews, 2010) was used to evaluate the quality of the raw Illumina data (approximately one billion paired‐end reads) and assess potential sample contamination. To estimate genome size, heterozygosity, and composition, the reads were put through the String Graph Assembler (SGA) version 0.10.15 PreQC module (Simpson & Durbin, 2010). Additional k‐mer analysis was run using Jellyfish k‐mer counter (Marçais & Kingsford, 2011) to create a k‐mer abundance plot. SGA estimated the genome size at 2706.3 Mbp, with a high abundance of low‐copy but k‐mer distinct repeats. Both Jellyfish and SGA displayed an extremely low level of heterozygosity.

Due to its large size, low heterozygosity, and large number of repeats, MaSuRCA v. 3.2.9 was selected as the assembler for constructing the reference genome. Default parameters for non‐bacterial Illumina assemblies were used except for a k‐mer count threshold of 2.0 (due to high coverage), insert size for pair‐end reads of 350 and standard deviation of 80, and an expected ploidy of 1.0 due to extremely low heterozygosity. Raw data were supplied without trimming, as MaSuRCA includes its own error correction module for which no trimming is generally recommended. Reads with median Phred score 20 or lower were, however, filtered. The resulting assembly had a total of 1,353,458 contigs, with an N50 of 2.8 Kb, and a total assembly length of 2113 Mb. Despite the high level of fragmentation, this compared favorably with the N50 of the best decapod genome available at the time, *Procambarus fallax f*. *virginalis*, which was under 2 Kb.

### 2.4 Generation of Genotyping‐By‐Sequencing markers

Numerous methods were trialed for extracting genomic DNA from kōura pleopods, which are small, tough, and rich in pigments (Li et al., 2011). Ultimately, a CTAB‐phenol‐chloroform method adapted from Panova et al. (2016; see Supplementary Information for details) proved most effective. All samples were quality‐tested (Nanodrop and agarose gel electrophoresis) and quantified (Qubit) prior to GBS library preparation. GBS data were generated from two separate libraries following the Elshire et al. (2011) method using 100 ng of genomic DNA and 1.44 ng of total adapters. The genomic DNAs were digested with the PstI restriction enzyme, and the library was amplified with 18 PCR cycles. Because samples were collected over the course of 2 years, library preparation and sequencing were completed in two separate batches. Both batches were sequenced with paired‐end, 2 × 150 bp reads on one lane of an Illumina X Ten through Custom Science, Ltd. To assess batch effects (i.e., library and lane biases; Leigh et al., 2018), 15 individuals were represented in both batches to ensure similar genetic distance estimates were produced by each duplicated sample independently.

SNP discovery and filtering were performed using both batches as a single dataset to better enable comparisons of genetic diversity and inbreeding between populations (Schmidt et al., 2021). Raw Illumina data were evaluated with FastQC v0.11.9 (Andrews, 2010) to assess quality and contamination levels. Paired‐end reads were demultiplexed with Axe v0.3.3 (Murray & Borevitz, 2018) with ‐m set to 0 (i.e., no adapter mismatches allowed) and GBS barcodes trimmed with Moraga's GBS Pre‐Process script (https://github.com/Lanilen/GBS‐PreProcess) which used Trim Galore v0.6.6 (https://github.com/FelixKrueger/TrimGalore) and Cutadapt v.3.1 (Martin, 2011). When using pair‐end sequencing, GBS forward and reverse reads overlap such that they contain essentially the same information (Rochette & Catchen, 2017). Thus, including both ends is unnecessary for SNP discovery, but their inclusion can increase confidence on SNP calls by doubling coverage at those sites. For this reason, the trimmed pair‐end reads were joined into single‐end files and subsequently aligned to the kōura short‐read reference genome with BWA‐MEM2 v2.1 using the single‐end setting (Vasimuddin et al., 2019).

Stacks v2.54 was used to discover and genotype SNPs using the *refmap*.*pl* wrapper (Rochette & Catchen, 2017; Rochette et al., 2019). Briefly, *gstacks* was run with default parameters except maximum soft‐clipping level (set to 0.01 read length). The *population* program was run with parameters ‐p 1 and ‐r 0.1 (i.e., loci were only required to be present in single population and at least 10% of individuals per population to be processed). Loci produced from the same restriction enzyme cut site were also merged (‐‐merge‐sites).

SNPs were filtered for missing data and statistical bias in VCFtools v0.1.16 (Danecek et al., 2011). Filtering followed an iterative approach developed by O’Leary et al. (2018) to retain as many SNPs as possible (Table 1). After initially filtering for maximum genotype missingness of 0.70 and removing individuals with over 50% missing data, the dataset was filtered to a minimum depth of three and maximum mean depth of 150. Minor allele count (MAC) was set at two to reduce random error without sacrificing detection of low‐frequency variants (Linck & Battey, 2019). The dataset was then further filtered for maximum genotype missingness of 0.95. BCFtools v1.11 (Li et al., 2009) was used to prune for linkage disequilibrium with the *r^2^* set to 0.6 and a window size of 1000 sites. Finally, individuals with greater than 10% missing data were removed to minimize bias when assessing genetic diversity and population genetic structure (Larson et al., 2021; O’Leary et al., 2018; Yi & Latch, 2021).

**TABLE 1:** SNP filtering workflow including the number of residual genomic sites (Sites) and individuals (*n*). Filtering was performed with VCFtools, BCFtools, and STACKS populations

Filtering procedure	*n*	Sites
Raw VCF from *populations* (STACKS2)	194	2,137,402
Minimum mean depth 10 Maximum missing genotypes 50% Individual missingness below 90%	186	166,725
Maximum missing genotypes 60% Individual missingness below 70%	180	163,369
Maximum missing genotypes 70% Individual missingness below 50%	177	156,972
Minimum depth 3 Maximum mean depth 150 MAC 2	177	47,070
Maximum missing genotypes 90% Individual missingness below 40%	171	9729
Maximum missing genotypes 95% Individual missingness below 25%	170	3630
Remove SNPs with LD r2 > 0.6 in 1000 site window	170	3235
Individual missingness below 10% (biallelic sites)	159	3188

### 2.5 Place‐based knowledge of kōura movement

Place‐based knowledge of kōura enabled *a priori* hypotheses around potential phylogeographic discordance and/or genetic admixture between sites. Ancestral Kāi Tahu narratives describe translocation of species such as kōura and tī kōuka (cabbage tree) along traditional pathways, often seeding new populations or augmenting existing ones as part of tribal economies and to provide sustenance on the way (e.g., Te Pae Kōrako, 2017).

Kāi Tahu developed many traditional routes to link settlements and mahika kai resources from the mountains to the coasts… Our tīpuna (ancestors) travelled widely, following seasonal food sources around Te Waipounamu, hunting and gathering animals, plants and marine life. (Tamati‐Elliffe)

Post‐European arrival, miners and contemporary hunters from local communities also translocated kōura, including into isolated catchment heads or fire ponds to be retrieved for food on the way home.

A forester told me about a night where he released buckets of Central Otago kōura into the North Otago coastal Herbert Forest ponds … I also spoke to an angler [fisherman] with a bucket of kōura who was intending to release them in every creek between Cromwell and Queenstown—there is a population in Arrowtown that is in a really odd location! Past glacial action would have removed species from that region (Ferrar, 1928), and kōura are unlikely to migrate upstream through fast flowing rivers like the Kawarau. (Hollows)

While Kāi Tahu knowledge of, and relationships with, mahika kai persist, colonization has eroded many details around pre‐European translocation of southern kōura. However, some recent translocations are more readily recounted. Murihiku populations M1 and M6 were translocated from surrounding forest streams in 2012 and 2008, respectively, and selectively harvested since then to enhance growth rates (Hollows, pers. comm.). Kōura in lake W1—which lies adjacent to traditional travel routes and is a popular site for catching trout today—were translocated from elsewhere (McDowall, 2005). Lake W3 is also a known site of translocation, with the surrounding forests “*renowned for hunting and kōura* … *hunters travel vast distances for the chance to find a big boar and a stag”* (Hollows, pers. comm.).

### 2.6 Estimating genetic diversity and population genetic structure

Because we anticipated unusual population genetic structure in kōura (Section 2.5), we used a range of genetic diversity and population genetic differentiation measures to provide greater confidence, especially for future management decisions (Zimmerman et al., 2020). We estimated genetic diversity with allelic richness, mean observed and expected heterozygosities calculated using _HIERFSTAT_ (Goudet, 2005) in R v4.0.2 (R Development Core Team, 2015). Private allele counts were also evaluated in the STACKS *populations* program (Rochette et al., 2019). To assess population genetic structure, we applied three complementary approaches. Fixation indices (i.e., *F*‐statistics) were implemented in diveRsity v1.9.89 (Keenan et al., 2013) including overall means (presented with 95% margin of error) and hierarchical pairwise comparison of *F_ST_*. Statistical significance for pairwise *F_ST_* was tested in diveRsity using 1000 permutations. Because these measures rely on assumptions of Hardy–Weinberg and linkage equilibrium that are generally violated in small, isolated populations, we also used STRUCTURE, which is more robust to deviations from Hardy–Weinberg and linkage equilibrium (Falush et al., 2003; Pritchard et al., 2000). STRUCTURE was run through the wrapper program Structure_threader v 1.3.0 (Pina‐Martins et al., 2017) using a total of 3188 variant loci across 159 individuals. The number of clusters (K) was determined by running trials for 20 levels of K (K = 1 to 20) with 100,000 cycles of burn‐in (BURNIN = 100,000), 100,000 Markov chain Monte Carlo samples (NUMREPS = 100,000) and 10 trials at each level (Porras‐Hurtado et al., 2013). Best K was determined using the Evanno method (Evanno et al., 2005), CLUMPAK (Kopelman et al., 2015), and visualization in STRUCTURE HARVESTER (Earl & vonHoldt, 2012). To examine hierarchical substructure, we also ran STRUCTURE at three regional levels (Murihiku, Ōtākou, and Waitaha) using the same parameters for up to nine levels of K (K = 1 to 9). Populations were assigned to each region based on the results of global genetic population structure analyses and personal communications (Section 2.5). Third, we ran principal components analysis (PCA) after imputing missing data to provide confidence around *F_ST_*‐based estimates of population differentiation (Jombart et al., 2009).

### 2.7 Detecting signatures of local adaptation

We used RDA to explore signatures of local adaptation in kōura (Flanagan et al., 2018; Hoban et al., 2016; Kierepka & Latch, 2016). Compared with other widely used EAA, including other constrained ordination methods such as generalized linear models (GLM) or latent factor mixed models (LFMM), RDA‐based methods are well‐placed for detecting polygenic signatures of local adaptation in heterogeneous landscapes under moderate to strong selection (Forester et al., 2016, 2018). In addition, RDA requires no population genetic assumptions (e.g., Hardy–Weinberg equilibrium) and is effective for a wide range of sampling designs and population genetic structure (Capblancq et al., 2018; Jombart et al., 2009), although their power to produce low false‐positive and high true‐positive rates depends on the appropriate selection of relevant predictors and model parameters (Forester et al., 2016). There is a suite of hydroclimatic indices available in global and national databases that can serve as a starting point for exploring relationships between environment and genetic variation. A total of 13 environmental variables (Table S1) were extracted and evaluated in R, including nine bioclimatic variables from the WorldClim database (Fick & Hijmans, 2017) and four flow‐related variables from the Ministry for the Environment's national database (Booker, 2015). These variables were chosen for evaluation because they are readily available, of interest to research partners, and include hydroclimatic features previously identified for other freshwater species (e.g., Brauer et al., 2016; Harrisson et al., 2017). Past experiences indicate that water contaminants, land‐use change, predator presence, calcium concentrations, and refuge availability strongly influence kōura presence (Hollows, Kusabs, Rupene, Thoms, pers. comm.), but we were unable to secure consistent data for this study. Finally, populations known or suspected to be translocated or descended from translocated individuals—either from population genetic structure or personal communications (see Section 2.5)—were excluded prior to RDA.

Scripts for RDA (available at https://github.com/UC‐ConSERT/2021_EVA_Rayne_et_al) were adapted from Brauer et al. (2018), Capblancq and Forester (2021), and the vignette provided with Forester et al. (2018) using the VEGAN v2.5–7 package (Oksanen et al., 2013). A forward selection procedure was run to further remove nonsignificant (*p* > 0.001) environmental predictors using the *packfor* R package (Blanchet et al., 2008; Dray et al., 2009). Predictors with Pearson correlation coefficients over 0.70 were collapsed, including variables associated with site latitude and longitude to minimize false positive detections due to spatial structure. Variance inflation factor (VIF) analysis was used to minimize multicollinearity (VIF <10; Dyer et al., 2010; Zuur et al., 2010). Since RDA requires complete data frames, missing values were imputed with the most common genotype across individuals. To account for spatial population structure, PCA was performed on 1450 putatively neutral SNPs, i.e., the same dataset pruned using *pcadapt* (Luu et al., 2017) to identify and exclude outlier loci with q‐value >0.05. Variance partitioning using partial RDA was used to assess the independent contributions of hydroclimatic explanatory variables, neutral population genetic structure, and geography, with the most significant of these included as conditioning variables in the RDA (Table S2, Figure S1; Forester, pers. comm., Capblancq & Forester, 2021). Full model and marginal significance of each environmental predictor were assessed with 1000 permutations of the response data, and significant constrained axes were identified using 1000 permutations and a *p*‐value threshold of 0.05. Outliers were identified following the *rdadapt* function described in Capblancq et al. (2018), which uses a distribution of Mahalanobis distances estimated between each locus and the center of the RDA space given a certain number of axes (K). Mahalanobis distances were corrected for inflation factor (François et al., 2016) and transformed into *p*‐values using a chi‐squared distribution with K degrees of freedom (Luu et al., 2017). Candidate adaptive outliers were identified using a Bonferroni correction (i.e., returning a *p*‐value lower than 0.01/number of tests) to correct for multiple testing (Capblancq & Forester, 2021).

## 3 RESULTS

### 3.1 Estimates of genetic diversity

The final dataset included 3188 SNPs for 159 individuals sampled across 19 sites (Table 2). The mean observed global heterozygosity was significantly lower than expected global heterozygosity as per Bartlett's test of Homogeneity of Variances (mean *H_O_* = 0.040; *H_T_’* = 0.078; Bartlett's K^2^ = 712.92, df = 1, *p*‐value <2.2e^−16^). Within each population, expected heterozygosity per population (*H_S_*) was significantly lower than observed (Table 2). These patterns are reflected in fixation indices; namely, *F_IT_* indicates an overall deficiency of heterozygotes relative to the global population (*F_IT_* = 0.480 ± 0.025), but heterozygote excess within populations (mean *F_IS_* = –0.063 ± 0.025). Population genetic diversity generally decreased from Murihiku northward to Ōtākou and then Waitaha (Table 2), except for admixed populations such as W3 (see Section 2.5, above, and Section 3.2, below) M1 had the highest allelic richness (*α* = 1.067) and highest expected heterozygosity (*H_O_* = 0.067), while M4 contained the highest number of private alleles. Sites around Waitaha represented the least genetically diverse populations, the lowest of these being W4 (*H_S_* = 0.018), followed closely by W1 and W2.

**TABLE 2:** Descriptive genetic diversity statistics for 19 kōura populations including number of individuals (*n*), number of private alleles (Priv. all.), allelic richness (α), average observed heterozygosity (H_O_) ± standard error (SE), average expected heterozygosity per population (H_S_) ± SE, and deficiency of average heterozygotes (F_IS_) ± SE. Population abbreviations (Pop) correspond to sampling sites

Pop	*n*	Priv. all.	*α*	*H_O_* ± SE	*H_S_* ± SE	*F_IS_* ± SE
W1	7	34	1.021	0.024 ± 0.003	0.021 ± 0.002	−0.095 ± 0.012
W2	11	41	1.022	0.027 ± 0.003	0.022 ± 0.002	−0.081 ± 0.009
W3	12	48	1.050	0.045 ± 0.004	0.051 ± 0.003	0.206 ± 0.014
W4	7	29	1.019	0.021 ± 0.003	0.018 ± 0.002	−0.089 ± 0.013
O1	9	40	1.038	0.039 ± 0.004	0.038 ± 0.003	0.002 ± 0.010
O2	9	45	1.04	0.045 ± 0.004	0.039 ± 0.003	−0.087 ± 0.009
O3	10	44	1.034	0.039 ± 0.004	0.034 ± 0.003	−0.091 ± 0.009
O4	11	25	1.029	0.032 ± 0.003	0.028 ± 0.003	−0.074 ± 0.009
O5	7	68	1.033	0.038 ± 0.004	0.032 ± 0.003	−0.140 ± 0.012
O6	9	26	1.031	0.035 ± 0.004	0.031 ± 0.003	−0.077 ± 0.010
O7	5	5	1.032	0.035 ± 0.004	0.032 ± 0.003	−0.087 ± 0.012
O8	9	19	1.032	0.036 ± 0.004	0.032 ± 0.003	−0.066 ± 0.010
O9	7	18	1.026	0.027 ± 0.003	0.026 ± 0.003	−0.005 ± 0.012
M1	10	157	1.067	0.069 ± 0.005	0.067 ± 0.004	−0.026 ± 0.009
M2	8	8	1.054	0.054 ± 0.004	0.054 ± 0.004	−0.005 ± 0.011
M3	7	114	1.056	0.056 ± 0.004	0.056 ± 0.004	0.002 ± 0.010
M4	8	170	1.046	0.048 ± 0.004	0.046 ± 0.004	−0.020 ± 0.012
M5	3	125	1.049	0.051 ± 0.005	0.048 ± 0.004	−0.094 ± 0.014
M6	10	91	1.038	0.039 ± 0.004	0.038 ± 0.003	0.003 ± 0.011

### 3.2 Measures of population genetic structure

**FIGURE 3:**
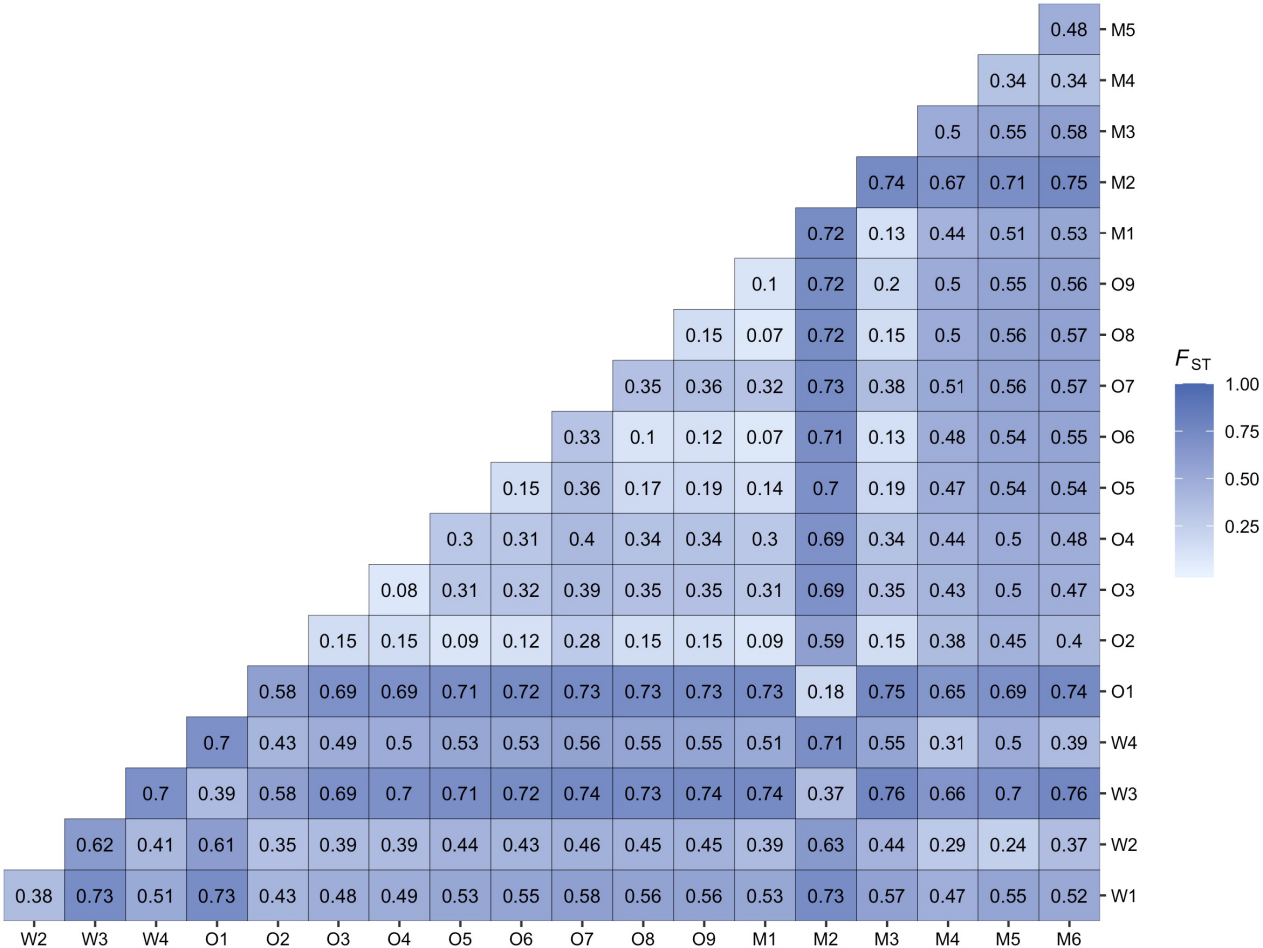
Heatmap showing pairwise FST (Weir and Cockerham's 1984 method) for 19 kōura populations distributed across Te Waipounamu

**FIGURE 4:**
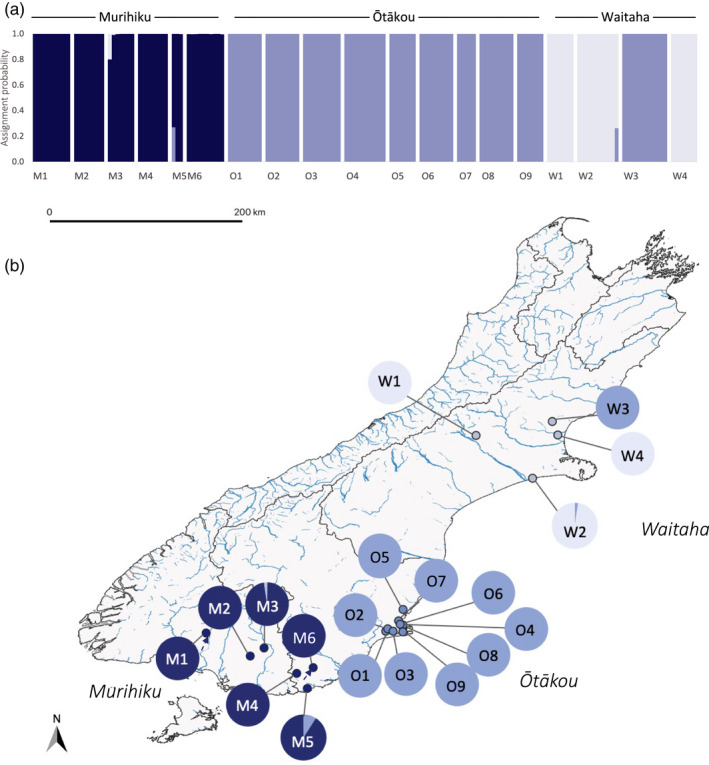
Assignment probabilities for 19 kōura populations distributed across Te Waipounamu produced by STRUCTURE when K = 3. (a) Each individual is represented by a vertical bar, with colors indicating the assignment probability to the Waitaha (light blue), Ōtākou (mid‐blue), or Murihiku (dark blue) cluster. (b) Mean of each population's individual assignment probabilities visualized on population distribution map as pie charts

**FIGURE 5:**
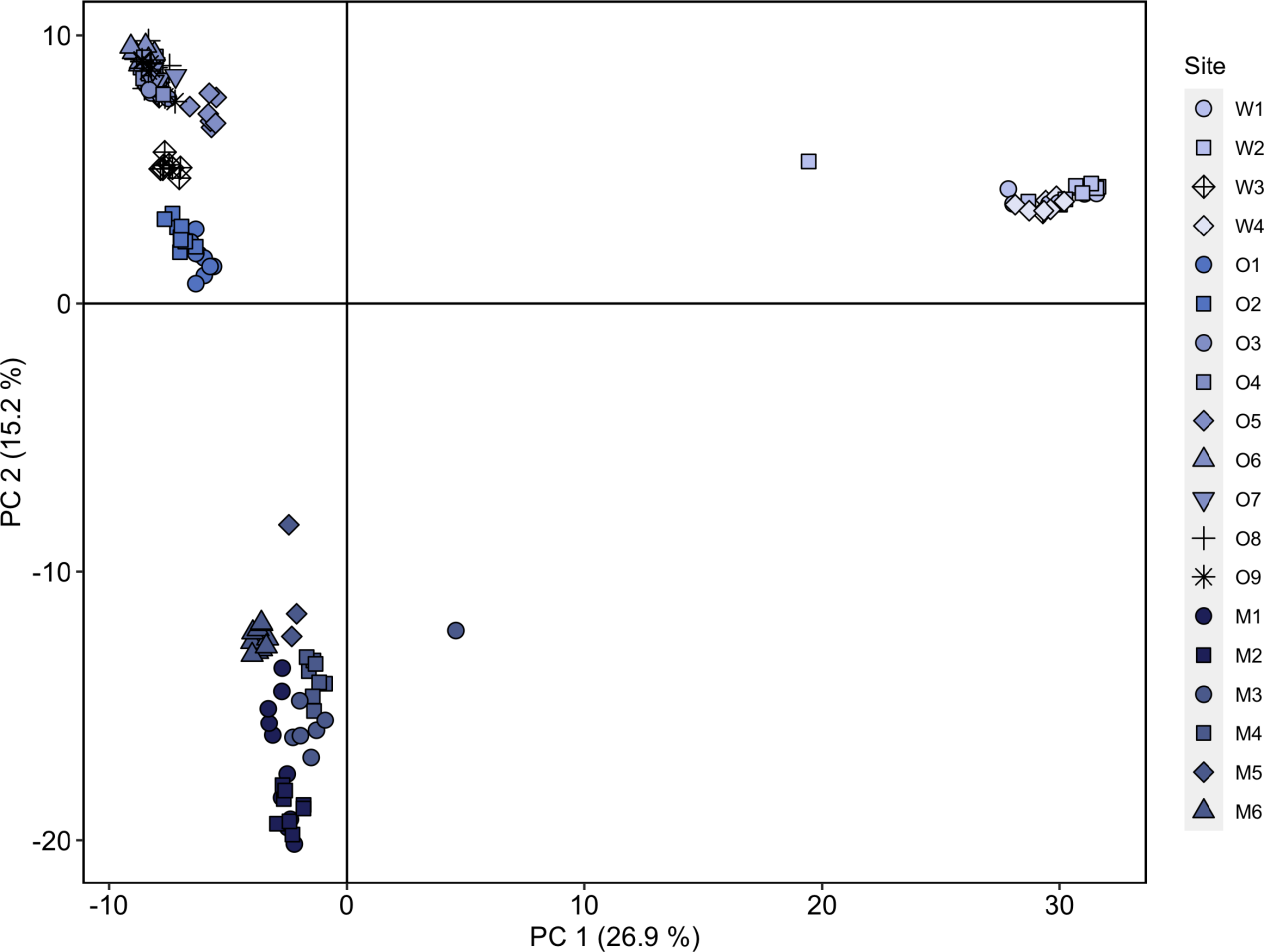
Principal components analysis of 19 kōura populations, color coded according to STRUCTURE assignments in Figure 6. Dotted lines in graph key delineate regions, with populations sampled from Waitaha in the top third of key, Ōtākou in the middle, and Murihiku in the lower third

Initial assessment of population genetic structure using Weir and Cockerham's (1984) estimate of *F_ST_* indicated strong and significant genetic structure across the entire distribution (global *F_ST_* = 0.511 ± 0.017). Pairwise *F_ST_*, which ranged from 0.07 to 0.76, also indicated strong and significant population genetic structure for all pairwise comparisons (Figure 3). STRUCTURE HARVESTER determined that the change in mean marginal likelihood probability of K (LnP(K)) was highest at K = 3 (Figure S2), corresponding to three geographic regions (Waitaha, Ōtākou, and Murihiku; Figure 4). All individuals were assigned to the geographic region from which they were sampled (assignment probability (Q) = 1.00), excluding three individuals with genetic assignment to more than one region (Table 3). Given the strong population genetic structure observed above, we used 0.95 as a conservative threshold for admixture. Thus, individuals with assignment probabilities <0.95 to a single cluster were considered admixed (Forsdick et al., 2021; Senn & Pemberton, 2009). PCA also supported regional population genetic structure identified by STRUCTURE (Figure 5). Notably, W3 falls between upper‐east and lower‐west Ōtākou subclusters, while the W2 and M3 individuals that fall further toward the middle of the PCA are the same individuals with admixture from Ōtākou and Waitaha, respectively.

**TABLE 3:** Sampling location (‘Site’), number of individuals per site (‘*n*’), primary and secondary assignment probabilities (Q) for individuals considered admixed (Q < 0.95) in the global STRUCTURE analysis

Site	*n*	Primary assignment	Secondary assignment
W2	1	Q_Waitaha_ = 0.74	Q_Ōtākou_ = 0.26
M3	1	Q_Murihiku_ = 0.80	Q_Waitaha_ = 0.20
M5	1	Q_Murihiku_ = 0.73	Q_Ōtākou_ = 0.27

**FIGURE 6:**
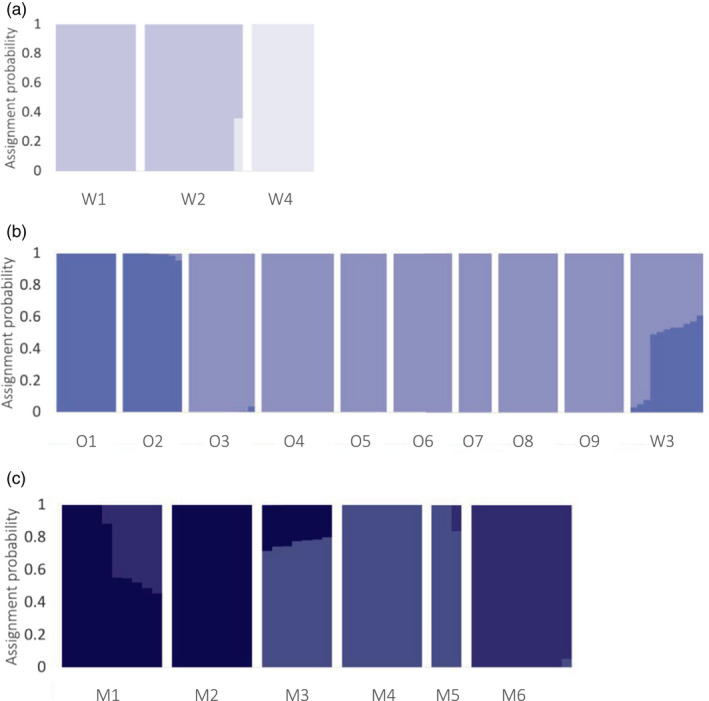
Assignment probabilities for kōura produced by STRUCTURE analysis at a regional scale. Each individual is represented by a vertical bar with colors indicating the assignment probability to each genetic cluster in (a) Waitaha (K = 2); (b) Ōtākou (K = 2); and (c) Murihiku (K = 3)

Given that all individuals in W3 were assigned to the Ōtākou cluster, the population was included in the Ōtākou—rather than the Waitaha—cluster for regional STRUCTURE analysis (Figure 6). The substructure was evident in all three regions. In Waitaha (*n* = 25 nloci = 531; maximal change in mean LnP(K) at K = 2; Figure S3), W4 emerged as a genetic cluster distinct from W1 and W2 (Figure 6a). For the Ōtākou sites, STRUCTURE revealed hierarchical substructure (*n* = 76, nloci = 1255; maximal change in mean LnP(K) at K = 2; Figure S4) between the lower‐west and upper‐east sampled region (Figure 6b). The substructure was also observed between the Murihiku sites (*n* = 46, nloci = 1942; maximal change in LnP(K) at K = 2; Figure S5). LnP(K) further increased from K = 2 to K = 3 (Figure S5a), and after further assessment with CLUMPAK (Kopelman et al., 2015) and PCA (Figure 5), we used K = 3 which identified M1 to M2, M3 to M5, and M6 as distinct clusters (Figure 6c). Given strong F_ST_ within regions (Figure 3), we again applied the Q < 0.95 threshold to identify admixture which ranged from zero to 12 individuals per population (Figure 6). The admixed individuals in W2 and M5 are the same two individuals identified in the global analysis (Table 3).

### 3.3 Weaving place‐based knowledge with exploratory analyses for local adaptation

Place‐based knowledge (Section 2.5) and population genetic structure (Section 3.2) identified three populations (M1, M6, and W3) and three further individuals (from W2, M3, and M5) known or suspected to be translocated or descended from translocated individuals. In line with Hollows (pers. comm.), genetic clustering and admixture inferred from STRUCTURE and PCA suggest that there were multiple translocations of kōura into W3 from sites in northern Ōtākou and Taiari catchments, several hundred kilometers away. The genomic markers also showed admixture within M1, and later discussion with co‐author John Hollows revealed that kōura from M6 had been introduced during the population's recent history. The history of admixed population M3, located at a popular camping and walking site, is not yet known (Parata, pers. comm.), as are the details around genetic introgression from Ōtākou in two individuals from W2 and M5, respectively.

To avoid reducing the power of RDA to detect associations between outlier loci and environmental predictors, we excluded these populations and three individuals (Table 3) from further analysis. Thus, the final RDA model included 118 individuals genotyped at 2606 SNPs. Forward selection and VIF analysis for multicollinearity produced a final set of five environmental predictors for RDA: altitude (Alt), average stream flow rate (AvFl), flow seasonality (Feb), annual precipitation (P), and temperature seasonality (TS). The first two axes of a PCA run on 1450 putatively neutral SNPs were retained as conditioning variables after assessing collinearity among the first six principal components, environmental predictors, and geographical coordinates (Figure S6). This partial RDA model was globally significant (*p* < 0.001) and indicated that the retained environmental predictors collectively explained 8.71% (adjusted R^2^ = 6.82%) of the total genetic variation after accounting for population structure, which explained 47.3% of the total variation in the model. All five constrained RDA axes were significant (*p* < 0.001), although most of the constrained variations (portion of total genetic variation explained by the environment) were accounted for in the first (33.0%) and second (25.5%) axes. The first axis was primarily driven by precipitation and temperature seasonality, whereas the second was strongly associated with the mean annual flow and flow seasonality (Figure S7). We identified 387 SNPs with extreme associations (i.e., *p*‐values <3.84 × 10^−6^) with these first two axes (Figure S8) for a final dataset of 353 unlinked putatively adaptive loci.

**FIGURE 7:**
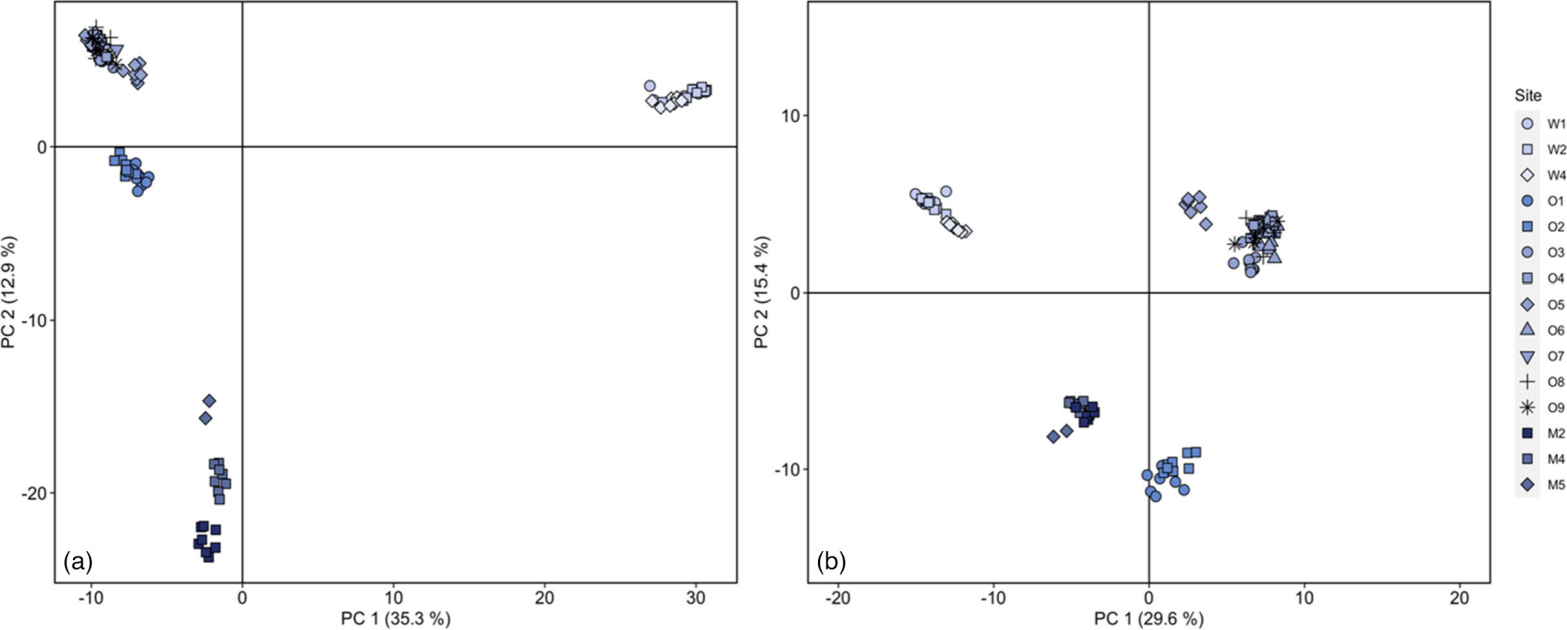
Principal components analysis based on (a) all loci (b) putatively adaptive loci only (rescaled for comparison). Both plots exclude two individuals and three populations known or presumed to be translocated or admixed (see text for details). Regional analyses are available in Supplemental Information (Figure S9)

To assess whether our putatively adaptive SNPs identified different population structure compared to genome‐wide (i.e., neutral and putatively adaptive) loci, we used PCA to reassess population structure using the 353 SNPs identified by RDA. These putatively adaptive SNPs identified similar groupings to genome‐wide loci but notably drew out W4, O5, and O3 as distinct groups and further separated O1 and O2 from the other Ōtākou populations (Figure 7). M2 and M5 also grouped together into a single adaptive ‘unit’ (Barbosa et al., 2018).

## 4 DISCUSSION

In this study, trusted partnerships between researchers and IPLC guided the framing of research, sampling co‐design, and interpretation of genomic markers. Place‐based knowledge was critical to enabling respectful sample collection, including through the identification of sites—particularly those not recorded in public databases—and selection of appropriate sampling methods (see Section 2.2). Discussions around past translocations (Section 2.5) also provided important biocultural context for interpreting population genetic structure, which allowed us to confidently adjust our analyses of local adaptation. Most importantly, our partnerships provided opportunities to reconnect with the environment and grow expertise among researchers, practitioners, and whānau, particularly youth. Our experiences also highlight the importance of prioritizing relationships over conventional academic timelines, especially when co‐developing genomic research for widely distributed species. It takes time to bring people together, to build trust, and for communities to decide whether and how to co‐produce and/or share knowledge. Moreover, many practitioners and whānau freely gave their time, knowledge, and energy to this study. Funding has since been secured for tribal‐led programs that include resourcing for relationship building and IPLC expertise (see Section 4.2).

### 4.1 Genomic markers reveal genetically depauperate but strongly genetically structured populations

Genomic markers indicated extremely low genetic diversity across 19 populations in the tribal territory of Kāi Tahu. Global heterozygosity was significantly lower than expected under Hardy–Weinberg equilibrium, as anticipated given the widespread decline and fragmented distribution of kōura. Within populations, higher than expected heterozygosity and absence of inbreeding by relatedness (i.e., low *F_IS_*) reflect patterns of global genetic erosion. In this scenario, the absence of large, outbred populations can distort allele frequencies and lead to erroneous signatures consistent with outbreeding (i.e., *heterozygote excess*) within highly related populations (Kardos et al., 2016; Keller & Waller, 2002). In short, genetic diversity primarily exists between—rather than within—kōura populations, leading to counterintuitive patterns of observed and expected heterozygosities.

Increasing genetic diversity from Waitaha to Murihiku aligns with phylogeographic evidence for south to east historical expansion of kōura (Apte et al., 2007) and place‐based knowledge of historical and contemporary movement of mahika kai species northward and inland from the coasts (Section 2.5; Hollows, Tamati‐Elliffe, pers. comm.). Strong population genetic structure indicated by PCA, STRUCTURE, and ubiquitously significant global and pairwise *F_ST_* lend further support toward low dispersal capacity and fragmentation in kōura. In Waitaha, particularly sparsely distributed and genetically depauperate populations (excluding site W3) may also reflect contemporary land‐use changes (Thoms, 2016). However, additional and historical genetic samples are needed to assess the extent to which these patterns reflect contemporary population declines or historical founder effects (e.g., McDowall, 2005). Untangling these demographic histories will be challenging, but with ongoing tribal‐led efforts to reconnect with place‐based knowledge and more comprehensive sampling, we anticipate future opportunities to explore the direction and timing of translocation events.

### 4.2 Weaving genomic markers with place‐based knowledge of past movement

The inclusion of different place‐based knowledge in this study enabled us to more confidently account for population genetic structure in tests for local adaptation. After excluding translocated populations, RDA identified 387 outlier loci linked to hydroclimatic variation—including precipitation, temperature seasonality, river flow rates, flow seasonality, and altitude—that were subsequently used to examine whether, and how, this putatively adaptive variation is shared among populations (Figure 7). Interpreted cautiously, these putatively adaptive loci can indicate relevant direct or indirect environmental pressures. Flow, for example, is recognized for its pivotal role in shaping eco‐evolutionary processes in river ecosystems over time by influencing geomorphology, sediment, habitat, food resources, and water quality (Elosegi et al., 2010). However, the hydroclimatic predictors included in our RDA models are coarse and may not capture fine‐scale variation that may be most relevant in shaping local adaptation, particularly in dynamic riverine systems. These models also exclude direct measures of abiotic or biotic variables (e.g., predator presence, water quality, and surrounding land‐use changes) known to profoundly impact kōura (Kusabs, Rupene, Thoms, pers. comm.). Among other limitations, including population genetic structure as a conditioning variable (i.e., to reduce the risk of false‐positive detections) could further conceal relevant signatures of local adaptation (Gibson & Moyle, 2020).

In the future, the inclusion of fine‐scale environmental predictors informed by place‐based knowledge, spatial structure (e.g., waterway distance and physical barriers) that better reflects waterscape heterogeneity (Davis et al., 2018), and more comprehensive genomic sampling may refine our understanding of the relative importance of hydroclimatic variables for kōura. Further, with a PacBio reference genome underway and overseas efforts to sequence the American lobster (*Homarus americanus*) genome, we anticipate future opportunities to explore signatures of local adaptation that incorporate transcriptomic, phenotypic, and experimental data. In the interim, the “holistic” nature of ordination analyses provide a useful starting point for understanding local adaptation in kōura (Capblancq et al., 2018; Steane et al., 2014) that can be combined with place‐based knowledge to inform management, for example, by identifying populations with distinct adaptive variation (e.g., Figure 7; Barbosa et al., 2018) and by prioritizing source populations for translocation that share similar historical water flow regimes to recipient sites (Hanson et al., 2020).

Our findings reiterate that researchers should be attentive to misleading signals of local adaptation in genomic data, particularly for populations with complex biocultural histories (e.g., culturally significant species that have been moved around in the recent or distant past) or in rapidly changing environments (e.g., due to range shifts or habitat fragmentation; Brauer & Beheregaray, 2020). Here, individuals or populations known or suspected to have experienced recent translocations (e.g., W3, M1, and M6) were excluded to minimize noise (i.e., decoupling of genotypes and ecotypes) that may have reduced the power of RDA to detect true signatures of selection. We anticipate this may be worth considering for other species with complex demographic histories or undergoing rapid range shifts, unless their previous distribution is thoroughly understood.

### 4.3 Next steps: informing place‐based strategies to meet intergenerational aspirations

Measures of genetic diversity and population genetic structure in threatened species are complex and context‐dependent, and these complexities become most salient in conservation decisions (Liddell et al., 2021; Love Stowell et al., 2017). For example, whereas temporal and spatial changes in biodiversity are often well‐characterized by IPLC (e.g., Bond et al., 2019; Herse et al., 2020), estimating the loss of diversity through genetic or genomic‐based measures can be challenging without contemporary or historically outbred populations for reference (Grueber et al., 2008). Nonetheless, our genomic markers indicate that each kōura population carries many private alleles and represents a significant and irreplaceable source of genetic variation. Moreover, each population is uniquely connected to different people and places. We anticipate strategies that incorporate a breadth of worldviews and metrics of biocultural diversity—including neutral and putatively adaptive genetic variation—will best equip kōura to persist in the face of change.

Today, many of these populations are on the brink of extirpation or vulnerable to urban or rural water contamination, flooding, and introduced predators. To protect their whakapapa, we are beginning to co‐develop mahika kai strategies, including conservation translocations, that will iteratively incorporate genomic and non‐genomic data. For example, moving kōura from the rapidly declining W4 population into nearby Tūhaitara Coastal Park could preserve the whakapapa of this important mahika kai population and simultaneously provide insights for best practices through monitoring co‐led by Ngāi Tūāhuriri (see Rayne et al., 2020). Translocation strategies should consider sourcing from multiple sites to minimize inbreeding and to avoid depleting natural populations (Mitchell et al., 2021). Preferentially translocating within “adaptive units” may also help to maintain local adaptations (Barbosa et al., 2018). Despite limited options for population augmentation within Waitaha, we are co‐developing plans to trial genetic rescue of population W4, preferably by initially sourcing individuals from population W2 (Figure S9b). In Ōtākou, we recommend that initial translocations into Te Nohoaka o Tukiauau (also see Rayne et al., 2020) start with source populations that group closely in Figure 7 and have historically shared the same catchment or similar catchments in close proximity (e.g., O1 and O2 in the Taiari catchment). In contrast to neutral genetic structure, putatively adaptive genomic markers also suggest that population such as M2 and M4 could be mixed without disrupting potential local adaptations. However, translocation strategies are ultimately determined by multiple considerations, including community relationships, practicability, and resourcing (Armstrong et al., 2019). For example, opportunities to express reciprocity and strengthen relationships between whānau and papatipu rūnaka may be prioritized over maintaining putative local adaptations when moving kōura within or between tribal regions.

To date, the research team has started conversations to co‐develop more comprehensive sampling and translocation strategies that support diverse aspirations for people and the environment (i.e., the final step of Figure 1). For example, some whānau are interested in understanding the whakapapa of populations in relation to ancestral travel routes. Many whānau and research partners from local communities are co‐developing strategies to enhance mahika kai or commercial values such as size, productivity, or color. To this end, our partnerships have been extended into several tribal‐led programs. In the short term, we anticipate these programs will strengthen relationships within and between tribal groups and partner organizations (e.g., through intergenerational transmission of knowledge and by growing capability of Māori and non‐Māori researchers to support tribal aspirations). Our long‐term vision is to build intergenerational capacity to restore the health of the environment, grow future generations as leaders and researchers, and revitalize sustainable harvest of kōura and other mahika kai species.

The distribution of kōura across multiple tribal territories and local communities also presents an important opportunity to co‐develop approaches that realize data sovereignty and benefit sharing in culturally significant or commercially sensitive genomic resources (Collier‐Robinson et al., 2019). For example, the data generated in this study are currently held in a locally managed, password‐protected data repository approved by research partners. We will continue to revisit data storage and access over time, especially given growing international initiatives to maintain Indigenous rights and interests in genomic resources and other culturally significant data (e.g., Anderson & Hudson, 2020; Liggins et al., 2021). As papatipu rūnaka and other IPLC continue to build capacity, this may include future opportunities to support or use tribally designed, owned, and managed repositories.

## 5 CONCLUDING REMARKS

It is now more critical than ever to recognize and reconnect with knowledge and relationships of place (Artelle et al., 2018; Des Roches et al., 2021; M’sɨt No’kmaq et al., 2021). Interdisciplinary genomic approaches (e.g., Aitken & Bemmels, 2016; Gougherty et al., 2021; MacLachlan et al., 2021) promise to help biodiversity thrive in the face of rapid change. However, without biocultural context, genomic data represent missed opportunities for IPLC and researchers to realize shared benefits, including the co‐creation of more nuanced and epistemically diverse knowledge (e.g., this study; Gros‐Balthazard et al., 2020; Henson et al., 2021; Polfus et al., 2016; Service et al., 2020). In particular, our experiences highlight the importance of partnership with multiple communities who use space in different ways. Such trusted partnerships can inform the benefits and risks of actions such as assisted gene flow or genetic rescue (Bond et al., 2019; Herse et al., 2021; Ralls et al., 2020; Service et al., 2014), and support intergenerational transmission of knowledge and practices, such as long‐term monitoring, through reconnection of people and place (e.g., Morishige et al., 2018). Whether, and how, such knowledge is shared remains up to the relevant IPLC (Black, 2014; Kimmerer, 2013; Mead, 2016; Roht‐Arriaza, 1996).

By providing a meeting place for place‐based knowledge outside “black‐box” academia, co‐created research can further draw attention to researcher positionality. Few Western‐trained researchers in ecology and evolution disclose the people and places they are connected to, or the “*assumptions*, *motivations*, *and value*s” that guide their research (Smith, 2013). We are confident that more explicit reflexivity, whether in personal communications or publication, will provide a stronger foundation for partnerships and create space for new approaches in genomic research (Beck et al., 2021). Finally, any partnership‐centered approach should include opportunities for IPLC to grow capacity and realize diverse needs and aspirations. For example, global initiatives such as the Summer internship for Indigenous peoples in Genomics (SING; www.singconsortium.org) and the FISHES project (Fostering Indigenous Small‐scale fisheries for Health, Economy, and food Security; http://fishes‐project.ibis.ulaval.ca/about‐fishes/) in northern Canada highlight opportunities to grow Indigenous leadership and address socio‐economic challenges. We look forward to seeing diverse Indigenous and local worldviews, expertise, and aspirations centered in future genomic research for culturally significant species.

## CONFLICT OF INTEREST

The authors declare no conflict of interest.

## Supporting information

Supplementary Material

## Data Availability

Filtering and RDA scripts are available on GitHub (https://github.com/UC‐ConSERT/2021_EVA_Rayne_et_al). Genomic data derived from taoka (treasured) species such as freshwater kōura are taoka (treasures) in their own right. Accordingly, the assembled short‐read reference genome for kōura, unfiltered GBS VCF, and associated metadata will be made available on recommendation of the papatipu rūnaka that affiliate as kaitiaki (guardians) for kōura from a local genome browser: http://www.ucconsert.org/data/.

## APPENDIX 1

### GLOSSARY

**Table:**

Aotearoa me Te Waipounamu	New Zealand
kēkēwai / kēwai / [wai] kōura	Freshwater crayfish, *Paranephrops* spp.
mahika / mahinga kai	Food gathering practices and places
maramataka	Māori lunar calendar
Māori	Indigenous Peoples of New Zealand
Pākehā	New Zealander, usually of European colonist descent
papatipu rūnaka / rūnanga	Local tribal groups with guardianship over land and water within their territory
rakatahi / rangatahi	Māori youth
tākata / tāngata tiaki	Guardians
Te Waipounamu	South Island of New Zealand
tīpuna / tūpuna	Ancestors
wānaka / wānanga	Learning forum, to meet and discuss
whakapapa	Genealogy
whakaweku	Bracken fern bundle
whānau	Extended family group
