# Supplementary material for: Weaving place‐based knowledge for culturally significant species in the age of genomics: Looking to the past to navigate the future
Source: Evol Appl. 2022 Apr 8;15(5):751–72. doi: 10.1111/eva.13367 (PMC9108313; doi:10.1111/eva.13367)
Supplement: Supplementary file 1 — Supplementary Material [file EVA-15-751-s001.pdf]

Supporting Information: Weaving place-based knowledges for culturally significant species in the age of genomics: Looking to the past to navigate the future

1 DATA SECURITY AGREEMENT FOR REFERENCE GENOME SEQUENCING 2

2 DNA EXTRACTION PROTOCOL FOR GENOTYPING-BY-SEQUENCING 3

3 SUPPORTING INFORMATION FOR GENETIC DIVERSITY AND POPULATION STRUCTURE ANALYSES 6

4 SUPPORTING INFORMATION FOR RDA ANALYSES 8

1 Data security agreement for reference genome sequencing

C | A | U Christian-Albrechts-Universität zu Kiel | Medizinische Fakultät

Institut für Klinische Molekularbiologie, Schittenhelmstr. 12, 24105 Kiel

Tammy Steeves, PhD
Senior Lecturer in Conservation Genetics
School of Biological Sciences
University of Canterbury | Te Whare Wānanga o Waitaha
Christchurch | Ōtautahi
New Zealand | Aotearoa

Directors
Prof. Dr. Stefan Schreiber
Prof. Dr. Andre Franke
Prof. Dr. Philip Rosenstiel

Rosalind-Franklin-Str. 12, 24105 Kiel
http://www.ikmb.uni-kiel.de

Prof. Dr. Andre Franke
Tel: 0431 / 597- 1098
Fax: 0431 / 597-2196
Email: a.franke@mucosa.de

Exzellenz im Norden

Inflammation at Interfaces

human development in landscapes

ozean der zukunft
DIE KIELER MEERESWISSENSCHAFTEN

26th[th] of September 2017

Dear Dr. Steeves,

Thank you for your interest in collaborating with us on your sequencing projects. We are aware of the cultural significance of your samples and would like to assure you and your collaborators that we will treat and protect any samples you decide to send to us with the appropriate respect and care. The Institute of Clinical Molecular Biology hosts one of the largest academic sequencing and genotyping facilities in Germany. As part of the University Hospital Schleswig-Holstein (UKSH), we have put special safeguards in place to ensure a high level of security - in compliance with national and European legislation for the handling of “sensitive” data (including, but not limited, to culturally significant genetic information).

Specifically, the following measures have been taken to eliminate or otherwise minimize concerns with regards to data protection:

- All sequencing facilities as well as primary data storage and processing facilities are located on premise; access to all facilities is strictly controlled
- Primary IT infrastructures are located inside the clinical network and are protected by its firewall
- Access to physical samples is only granted to authorized laboratory personnel and for the purpose of sample processing only
- Access to sequencing results is limited to authorized personnel strictly for the purpose of quality assurance and data delivery/archiving; no additional analysis of the data will be performed by us unless requested by the customer
- Samples are anonymized by the user prior to submission to our NGS facility; we are not able to assign samples to individuals on the basis of metadata available to us.
- Data is delivered to our users through a secure FTP (sFTP) server using password-protected, personalized accounts or, if desired, shipped on encrypted hard drives (e.g. VeraCrypt).
- After a project is completed, any remaining (physical) samples are returned to the customer. Regarding your project, we are happy to discuss with you an arrangement that meets your particular needs.
- In compliance with national regulations, all raw sequencing data is moved into our on-site archive storage where it is kept safe for a minimum of 10 years.

Should you require additional procedures to be adhered to, we are of course happy to discuss options and will try to accommodate your requirements where possible.

Best regards,

Andre Franke.

2 DNA extraction protocol for Genotyping-By-Sequencing

Adapted from [Panova et al. (2016)](#).

Note: β-Mercaptoethanol, phenol and chloroform are very toxic – these can be extremely harmful if inhaled or swallowed and may be fatal if absorbed through the skin. While working with these chemicals, wear protective gloves and perform all pipetting steps in a fume hood. When the tubes are taken out of the fume hood, their lids should be tightly closed. In the case of liquid spill on the surface of the tubes, wipe thoroughly with tissue, change gloves, and leave the tube in the fume hood until all smell disappears. Gloves, pipette tips, tissues, and plastic ware that have been in contact with β-mercaptoethanol, phenol or chloroform should be disposed of as hazardous waste.

Materials

1. SDS/CTAB buffer (1:1 mixture)[1].
2. β-mercaptoethanol.
3. Proteinase K (20 mg/mL).
4. Protease (7.5 AU, Qiagen).
5. RNase A (100 mg/mL).
6. Phenol:chloroform:isoamyl alcohol 25:24:1 (PCIA).
7. Chloroform:isoamyl alcohol 24:1 (CIA).
8. Isopropanol (2-Propanol).
9. Glycogen (5 mg/mL).
10. 70% ethanol, 0.1× TE buffer or nuclease- free water to dissolve the DNA.
11. Genomic DNA Clean & Concentrator-10 Kit (Zymo Research).
12. Phase Lock Gel Heavy 2 mL columns (PGL columns; 5 Prime).
13. Safe-Lock tubes 1.5 mL (Eppendorf).
14. Fume hood.
15. Incubator for 60 °C with a shaking platform (e.g., Heidolph 1000).
16. Mixer Mill (e.g., Retsch MM301 with stainless steel balls).
17. Centrifuge for 1.5 mL tubes with cooling capacity (4 °C) and maximum speed ≥ 18,000×*g*.
18. Tissue samples can be collected from living animals or animals stored in 96–99% ethanol. For fixation, tissues is placed in tubes with ethanol, and stored at 4 to −20 °C. To prevent DNA degradation and improve purity of DNA, it is important to change the ethanol twice, first 1 day and then 1 week after fixation.

---

[1] **CTAB buffer**: 2% w/v cetyltrimethyl ammonium bromide (CTAB), 1.4 M NaCl, 20 mM EDTA, 100 mM Tris–HCl pH 8.0, 0.2% v/v β-mercaptoethanol. For 100 mL, weigh 2 g of CTAB and 8.2 g of NaCl; add 70 mL of double-distilled water, 10 mL of 1 M Tris–HCl pH 8.0, and 8 mL of 0.25 M EDTA. Let the salts dissolve under magnetic stirring and bring the volume up to 100 mL with double-distilled water. The solution can be stored at room temperature for 6 months (CTAB will precipitate in the refrigerator).

SDS buffer: 0.7% w/v SDS, 10 mM NaCl, 10 mM Tris– HCl pH 8.0. For 300 mL buffer, add 2.1 g of SDS, 3 mL of 1 M Tris–HCl pH 8.0, and 6 mL of 0.5 M NaCl to 280 mL of double-distilled water. Let the SDS dissolve under magnetic stirring and bring the volume up to 300 mL with distilled water. The solution can be stored at room temperature for months.

Method

1. The day before the extraction, mix a 1:1 mixture of CTAB and SDS buffers and add 2uL of β-mercaptoethanol per 1mL mix (I prepare tubes with **200uL each of CTAB and SDS plus 0.8µL β-mercaptoethanol**). Incubate the mixture at **60 °C overnight** to dissolve the precipitate.
2. Prepare 1.5 mL Safe-Lock tubes with 400 µL of the digestion buffer (CTAB:SDS:β-mercaptoethanol), 20 µL of Proteinase K, and 20 µL of Protease (I used 40 µL Proteinase K, as we didn't have Protease on-hand). Place in the heating block at 60 °C.
3. Dissect pleopods (or tissue samples of approximately 2×2×2 mm size, avoiding the gut and exoskeleton) and place them directly upon dissection in the tubes with preheated digestion buffer.
4. Vortex samples well and incubate at 60 °C on the shaking plat- form for 1 h at 150 rpm (if an incubator with a shaking platform is not available).
5. Homogenize the tissue. I used clean plastic pestles for tail muscle tissue (i.e., intended for PacBio) to avoid shearing DNA. For pleopods (intended for GBS), I homogenised tissue using the Mixer Mill: add two steel balls per sample and shake for 5 min at 30 Hz/s.
6. Incubate for 30 min at 60 °C as in **step 4** to let the bubbles and foam formed during homogenization disappear and then (if using Mixer Mill) remove the metal balls used for homogenization with forceps or a magnet.
7. Add 5 µL of RNase A, vortex, and incubate for 1 h at 60 °C as in **step 4**.
8. Cool the samples for 5 min at room temperature. During this time, pre-spin the PGL columns (three per extraction) for 1 min at 14,000×*g* at room temperature.
9. Add 400 µL of PCIA, close the lids tightly and mix by inversion (either by hand or in a rotator) for 15 min at 40 rpm.
10. Pour the mixture into PGL columns and centrifuge for 5 min at 14,000×*g* at room temperature to separate the phases (if PGL columns are not available, phase separation can be done in microcentrifuge tubes).
11. Add 400 µL of PCIA into the same PGL column, close the lids tightly and mix by inversion for 10 min at 40 rpm.
12. Centrifuge for 5 min at 14,000×*g* at room temperature to separate the phases.
13. Pour the upper phase into a new PGL column, add 400 µL of CIA and mix by inversion as in **step 11**.
14. Centrifuge as in **step 12.**
15. Transfer the upper phase into a new PGL column, add 400 µL of CIA, and mix by inversion as in **step 11.**
16. Centrifuge as in **step 12.**
17. Transfer the upper phase into a new microcentrifuge tube.
18. Add 2 µL of glycogen and 400 µL of room temperature isopropanol. Mix by inverting slowly five times and incubate for 5 min at room temperature.
19. Centrifuge for 45 min at maximum speed (≥18,000×*g*) and 4 °C to pellet the DNA. At the end of centrifugation, there will be whitish DNA pellets visible at the bottom of the tubes.
20. Carefully decant or pipette out the liquid.

21. Add 1 mL of 70% ethanol (room temperature) and wash the pellets by inverting the tubes in the rotator for 5 min at 20 rpm.
22. Centrifuge for 5 min at 14,000×*g* at room temperature.
23. Carefully decant or pipette out the ethanol. It is not necessary to remove all the ethanol, just as much as can be removed without disturbing the DNA pellet. The pellets may float and it is important to make sure they are not lost during the washing steps. Repeat washing **steps 20** and **21** with 500 µL of 70% ethanol.
24. Remove all the ethanol: first decant as much liquid as possible and then spin briefly to collect the drops at the bottom and remove the rest using a 100 µL pipette.
25. Air-dry the pellets in the open tubes at room temperature. Avoid over-drying.
26. Add 50 µL of 0.1× TE buffer or nuclease-free water and let the pellets dissolve overnight at 4°C.
27. Mix gently by inverting and tapping the tubes (vortexing of the DNA solution may lead to shearing of the DNA), centrifuge briefly to collect the solution at the bottom of the tubes, and measure the DNA concentration.
28. Use the Genomic DNA Clean & Concentrator-10 Kit according to the manufacturer's protocol to further clean the samples. I tried the Zymo kit, however lost all my DNA – because I had limited tissue and time, I ended up sending DNA for sequencing without the clean-up step but it's worth experimenting if you can!

3 Supporting information for genetic diversity and population structure analyses

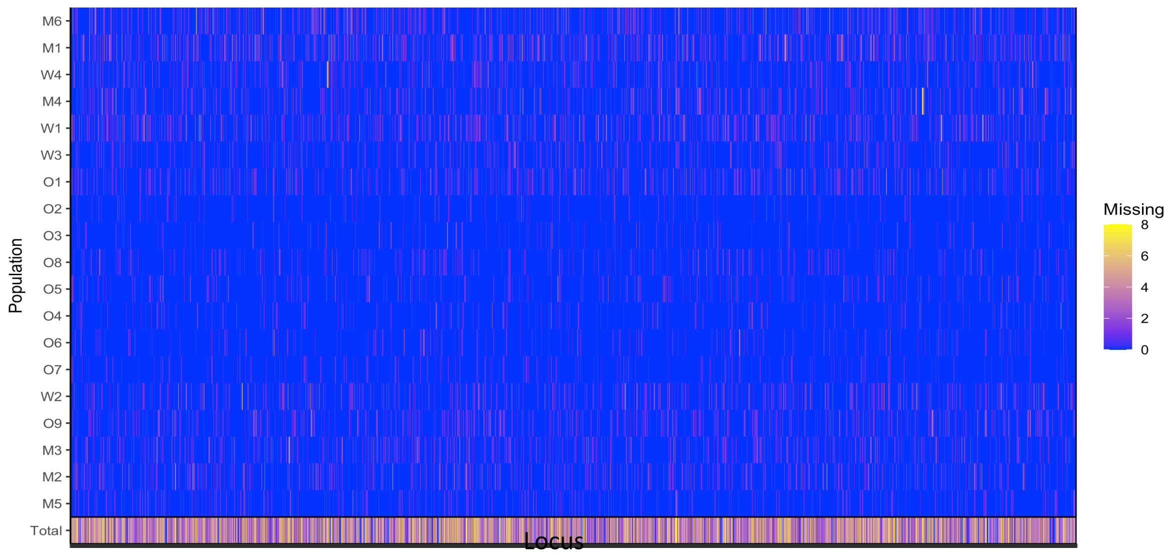

Figure S1. Plot summarising missing data across loci and populations (produced in poppr v2.9.1).

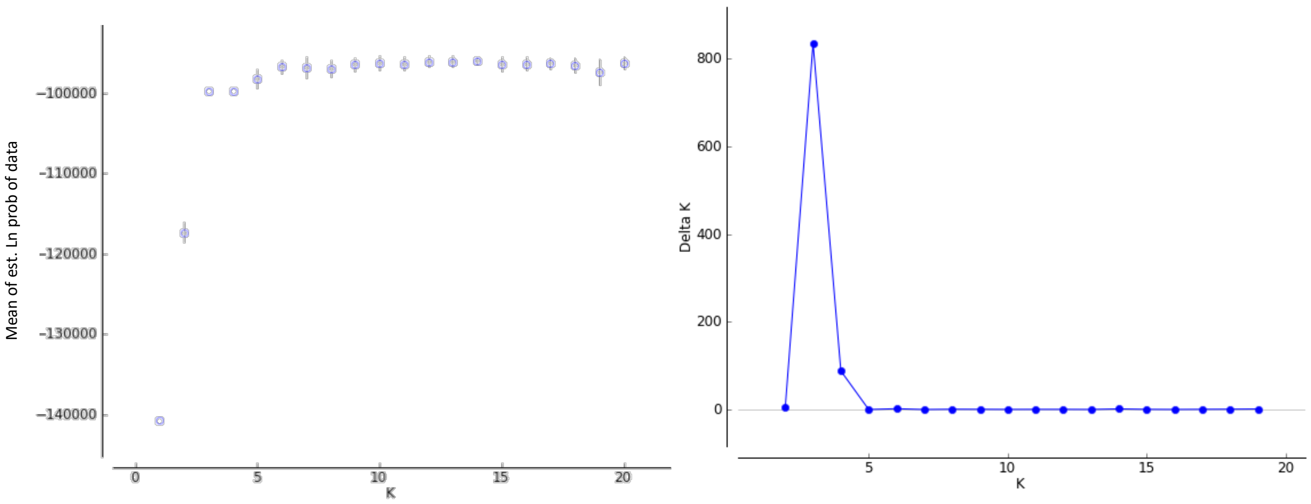

Figure S2. (**a**) Mean LnP(K) ± standard deviation and (**b**) change in LnP(K) for after 10 trials for each value of global K (burn-in period = 100,000, MCM reps = 100,000) performed in STRUCTURE, visualised here using STRUCTURE HARVESTER (**Earl & vonHoldt, 2012).**

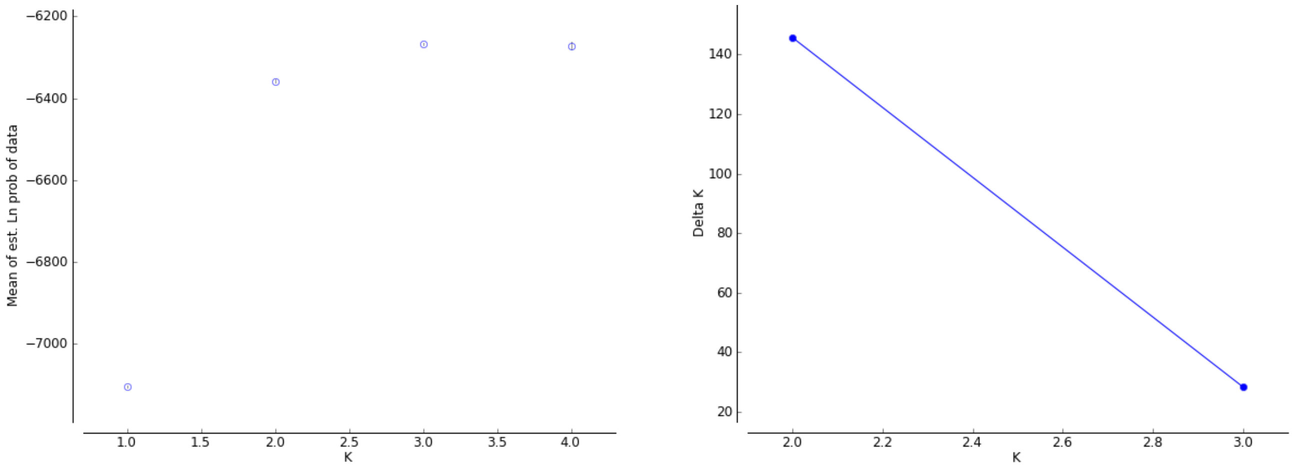

Figure S3. (**a**) Mean LnP(K) ± standard deviation and (**b**) change in LnP(K) for after 10 trials for each value of Waitaha K (burn-in period = 100,000, MCM reps = 100,000) performed in STRUCTURE, visualised here using STRUCTURE HARVESTER (**Earl & vonHoldt, 2012**).

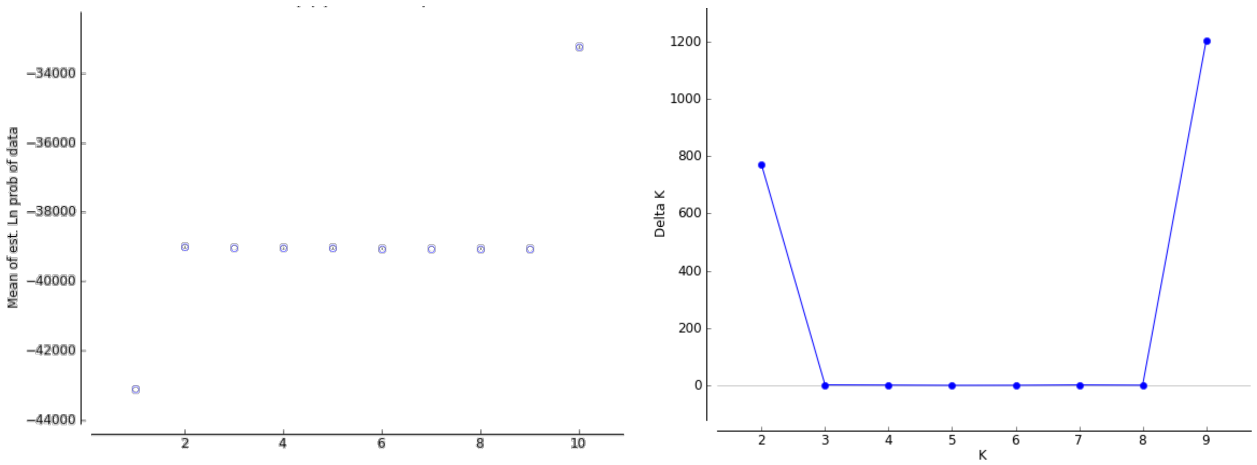

Figure S4. (**a**) Mean LnP(K) and (**b**) change in LnP(K) for after 10 trials for each value of Ōtākou K (burn-in period = 100,000, MCM reps = 100,000) performed in STRUCTURE, visualised here using STRUCTURE HARVESTER (**Earl & vonHoldt, 2012**).

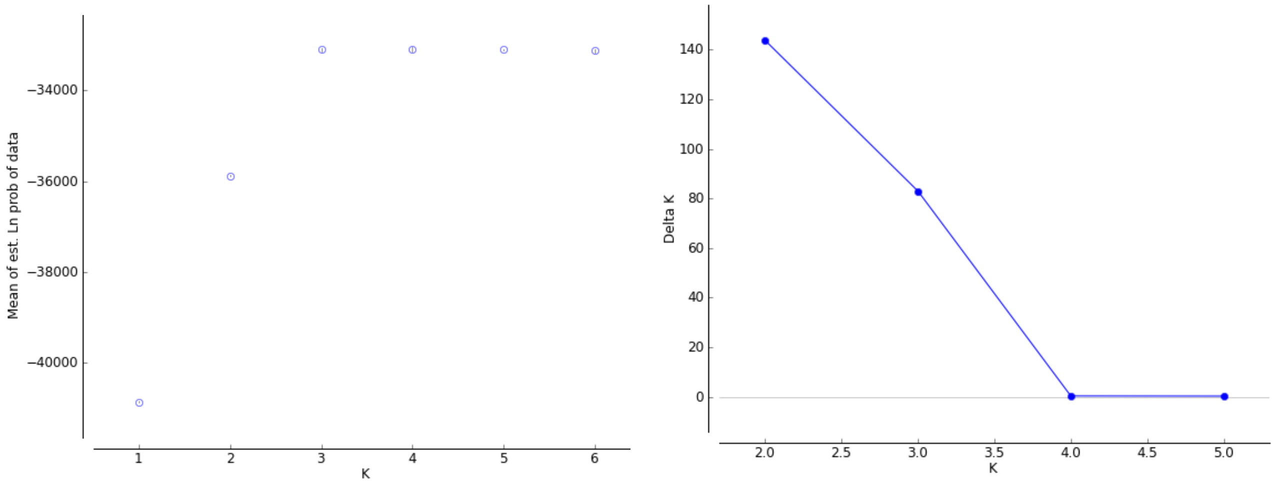

Figure S5. (**a**) Mean LnP(K) and (**b**) change in LnP(K) for after 10 trials for each value of Murihiku K (burn-in period = 100,000, MCM reps = 100,000) performed in STRUCTURE, visualised here using STRUCTURE HARVESTER (**Earl & vonHoldt, 2012**).

4 Supporting information for RDA analyses

Table S1. Hydroclimatic predictors evaluated in redundancy analysis (RDA) for 15 populations (Pop). Stream reach number (NZReach) and spatial coordinates (Lat, Long) not shown here to preserve anonymity of sampling sites. Bioclimatic predictors downloaded from WorldClim database include annual mean temperature (AMT), diurnal range (DR), temperature seasonality (TS), Max Temperature of Warmest Month (MaxT), min temperature of coldest month (MinT), mean annual temperature range (TR), annual precipitation (P), precipitation seasonality (PS) and altitude (Alt). Flow variables extracted from the Ministry for the Environment database include mean annual low flow (MALF), average flow (AvFl), mean flow in February divided by mean flow (Feb) and frequency of events exceeding three time the median river flow (FRE3).

Pop	NZReach	Long.	Lat.	AMT	DR	TS	MaxT	MinT	TR	P	PS	Alt	MALF	AvFl	Feb	FRE3
W1	–	–	–	89	92	3745	191	-8	199	1113	16	546	0.002	0.007	0.569	9.672
W2	–	–	–	117	94	3703	217	17	200	641	15	3	0.192	0.775	0.554	5.452
W4	–	–	–	120	93	3674	219	21	198	657	12	16	0.004	0.008	0.789	6.880
O1	–	–	–	103	81	3411	191	15	176	860	10	43	0.079	0.610	0.522	12.586
O2	–	–	–	95	84	3533	187	5	182	1056	10	134	0.013	0.079	0.571	13.204
O3	–	–	–	96	82	3461	186	8	178	1043	10	143	0.005	0.028	0.557	13.523
O4	–	–	–	105	81	3432	194	17	177	789	11	16	0.054	0.277	0.623	11.543
O5	–	–	–	100	86	3611	194	8	186	721	12	85	0.008	0.050	0.550	9.726
O6	–	–	–	97	82	3487	188	8	180	945	10	30	0.005	0.039	0.486	12.531
O7	–	–	–	97	82	3487	188	8	180	945	10	5	0.004	0.039	0.494	12.588
O8	–	–	–	101	80	3379	190	14	176	877	10	35	0.002	0.011	0.534	13.077
O9	–	–	–	104	79	3345	190	17	173	844	10	77	0.001	0.006	0.516	13.634
M2	–	–	–	90	96	3587	192	-6	198	1213	14	126	0.018	0.139	0.597	15.276
M4	–	–	–	82	92	3590	182	-11	193	1276	12	260	0.009	0.044	0.638	14.068
M5	–	–	–	98	88	3321	189	7	182	1195	13	11	0.002	0.011	0.528	14.193

Table S2. Adapted from Capblancq and Forester (2021). The influence of hydroclimate, geography, and neutral genetic structure on genetic variation decomposed with partial RDA. Inertia is analogous to variance. The proportion of explainable variance represents the total constrained variation explained by the full model.

Partial RDA models	Inertia	r^2	p (> F)	Proportion of explainable variance	Proportion of total variance
Full model: *F ~ hydroclim. + geog. + struct.*	226.4	0.707	0.001 ***	1.00	0.71
Pure hydroclimate: *F ~ hydroclim. | (geog. + struct.)*	15.1	0.047	0.001 ***	0.07	0.05
Pure structure: *F ~ struct. | (hydroclim. + geog.)*	45.4	0.142	0.001 ***	25.1	0.14
Pure geography: *F ~ geog. | (hydroclim. + struct.)*	3.8	0.012	0.001 ***	0.02	0.01
Confounded hydroclimate/structure/geography	162.1			0.66	0.51
**Total unexplained**	93.7				0.29
**Total inertia**	320.1				1.00

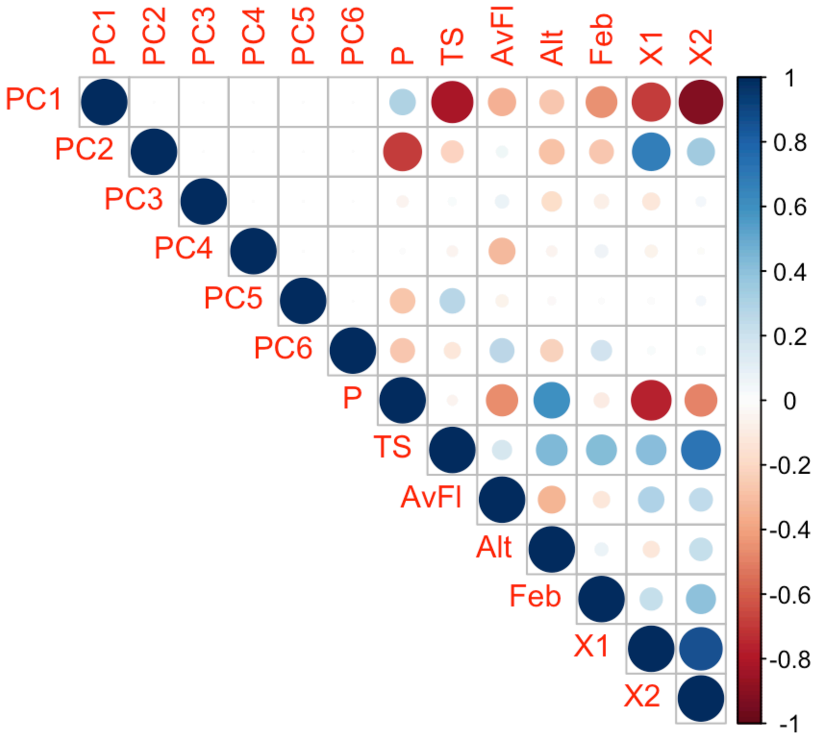

Figure. S6. Correlogram of the different variables estimated at each sampling site, including the nine selected climatic variables, the three first genetic PCs, longitude, and latitude. The most significant principal components axes and geographical coordinates were assessed for correlation (r) with environmental predictors and subsequently included as conditioning variables in the RDA where r > |0.7|

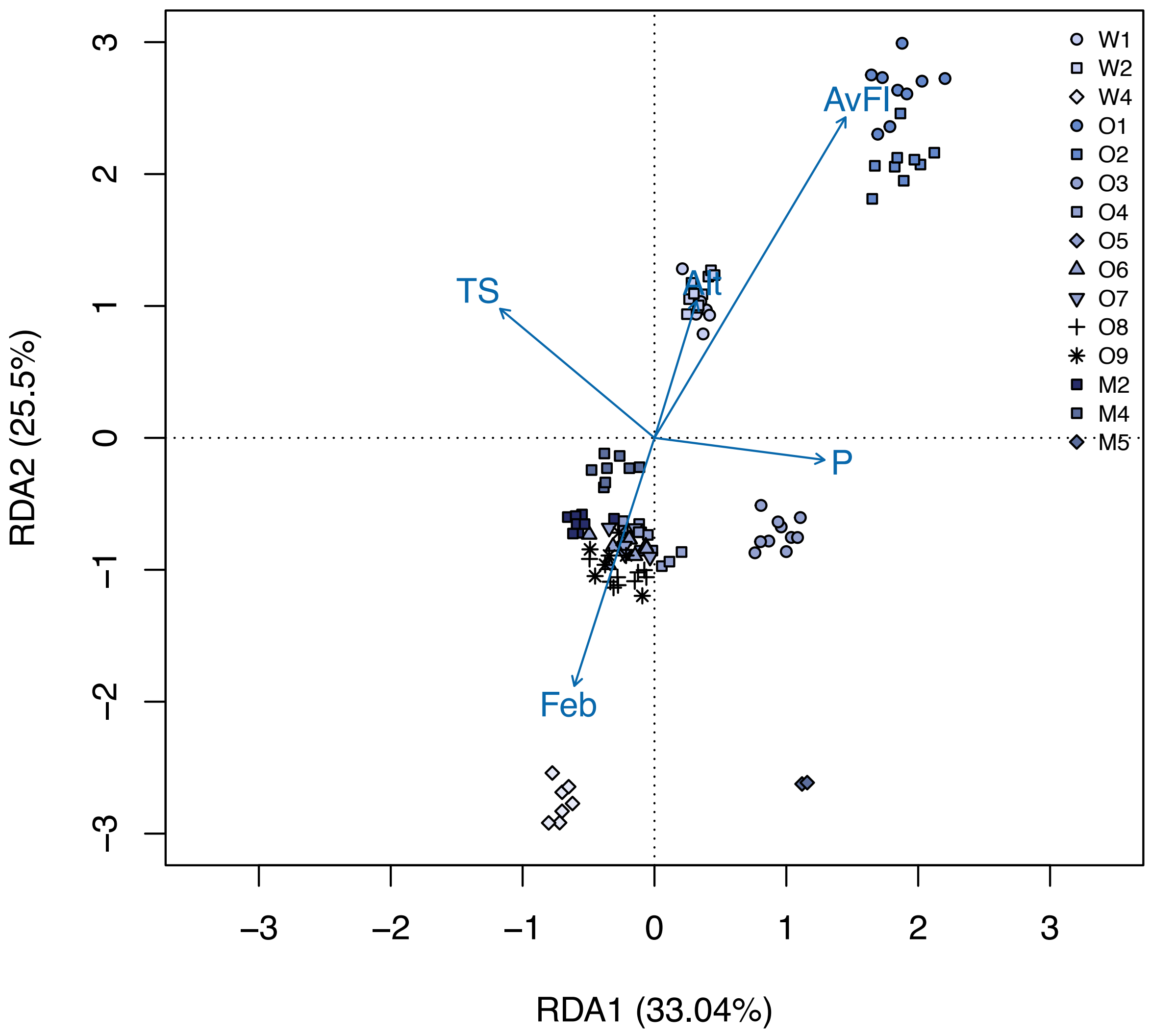

Figure S7. Triplots summarising the first two constrained axes of partial redundancy analyses (RDA) conditioned for spatial structure. Sampling sites are coded according to Fig. 5 and plotted based on site scores for each RDA. Six significant environmental factors ($p < 0.001$) related to altitude (Alt), average flow (AvFl), flow seasonality (Feb), annual precipitation (P) and temperature seasonality (TS) are represented as blue vectors where the length represents the magnitude of their contribution to the model and the angle between each vector represents the correlation among variables.

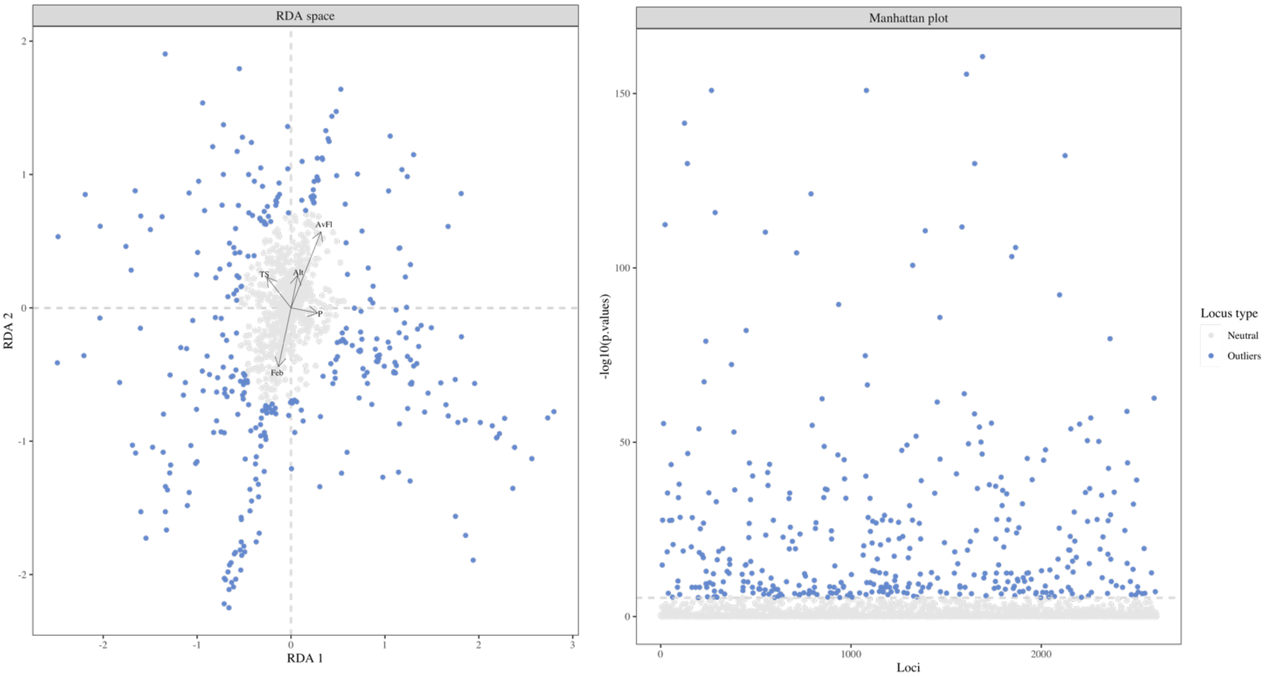

Figure S8. Results of the genotype-environment association using partial RDA. (**a**) The projection of loci and environmental variables along the first two RDA axes; **b**) A Manhattan plot with the distribution of -log10(p-values). Loci identified as outliers (p-value < 3.4 x 10-7) are coloured blue for both panels.

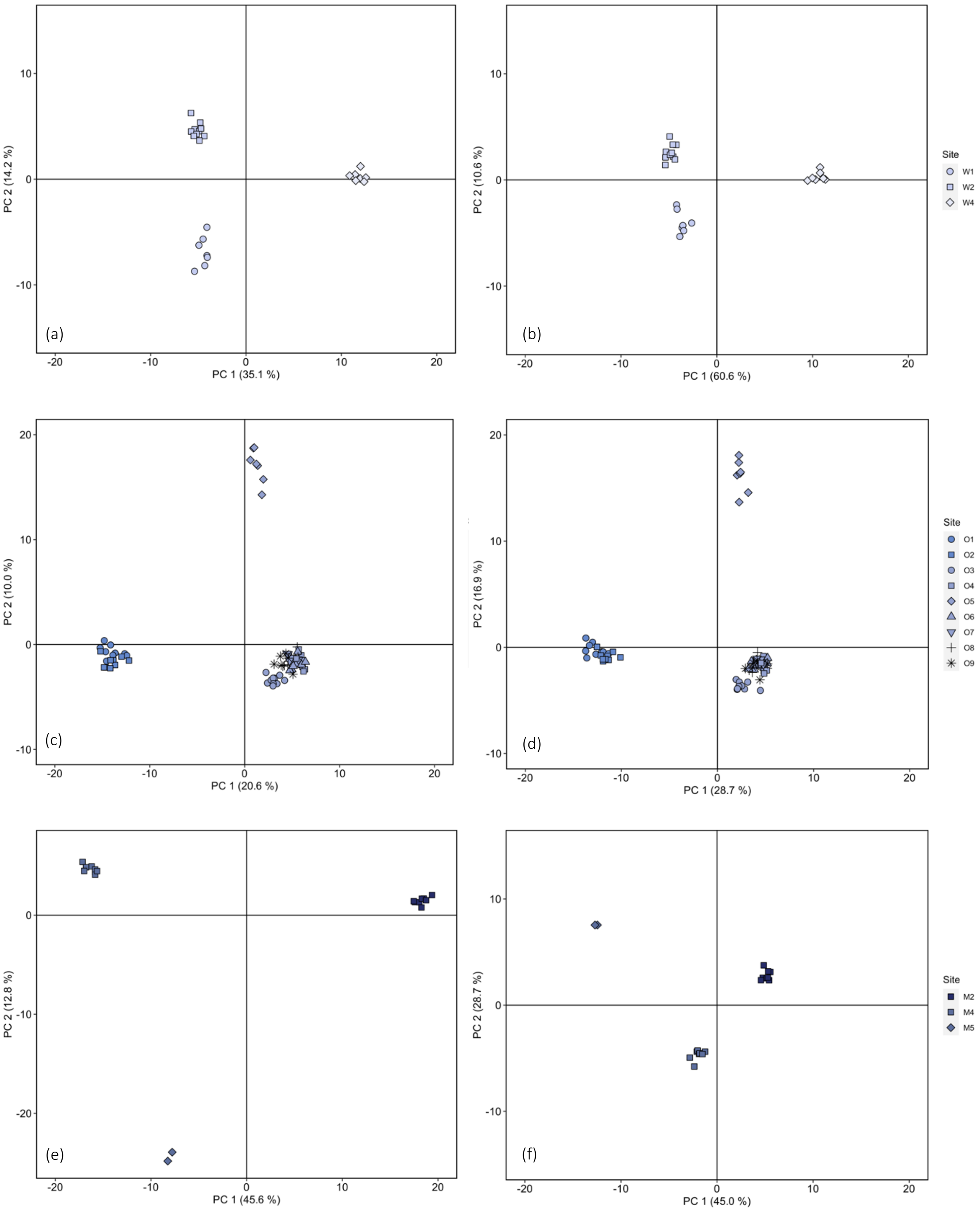

Figure S9. Regional comparison of principal components analysis based on all loci versus putatively adaptive loci only (rescaled for comparison). All plots exclude individuals and populations known or presumed to be translocated or admixed (see text for details). (**a**) Waitaha all loci; (**b**) Waitaha putatively adaptive loci only; (**c**) Ōtākou all loci; (**d**) Ōtākou putatively adaptive loci only; (**e**) Murihiku all loci; (**f**) Murihiku putatively adaptive loci only.
